# Assessment of an optimized manufacturing process for inactivated quadrivalent influenza vaccine: a phase III, randomized, double-blind, safety and immunogenicity study in children and adults

**DOI:** 10.1186/s12879-018-3079-8

**Published:** 2018-04-18

**Authors:** Carine Claeys, Mamadou Drame, José García-Sicilia, Khalequ Zaman, Alfonso Carmona, Phu My Tran, Mariano Miranda, Federico Martinón-Torres, Franck Thollot, Michael Horn, Tino F. Schwarz, Ulrich Behre, José M. Merino, Iwona Sadowska-Krawczenko, Henryk Szymański, Peter Schu, Elisabeth Neumeier, Ping Li, Varsha K. Jain, Bruce L. Innis

**Affiliations:** 1 GSK, Clinical Research and Development, Wavre, Belgium; 2 GSK, Clinical Evidence Generation (CEG), King of Prussia, PA USA; 3 Hospital Universitario HM Sanchinarro, Clinical Investigation in Vaccines Unit, Madrid, Spain; 4 icddr,b, MCHD Administration, Dhaka, Bangladesh; 5 Instituto Hispalense de Pediatría, Pediatría, Sevilla, Spain; 6 Cabinet Médical Tran, Pédiatrie, Nice, France; 7 Antequera Hospital, UGC de Pediatría, Málaga, Spain; 8 Department of Pediatrics, Santiago de Compostela, Hospital Clínico Universitario de Santiago, Translational Pediatrics and Infectious Diseases, Galicia, Spain; 9 Instituto de Investigación Sanitaria de Santiago and Universidade de Santiago de Compostela (USC), Grupo de Investigación en Genética, Vacunas, Infecciones y Pediatría (GENVIP), Galicia, Spain; 10 Association Française de Pédiatrie Ambulatoire (AFPA), Pédiatrie, Essey-les-Nancy, France; 11 Dr. med. Michael R. Horn Office, Pediatrics, Schoenau am Koenigssee, Germany; 12 Klinikum Würzburg Mitte, Standort Juliusspital, Central Laboratory and Vaccination Centre, Würzburg, Germany; 13 Ulrich Behre, Practice, Kehl, Germany; 14 Pediatric Department, Hospital Universitario de Burgos, Burgos, Spain; 15 Department of Obstetrics and Gynecology, Faculty of Medicine, Nicolaus Copernicus University in Torun, Collegium Medicum in Bydgoszcz, Torun, Poland; 16 Department of Neonatology, University Hospital No 2, Bydgoszcz, Poland; 17 NZOZ, Praktyka Lekarza, Oborniki Śląskie, Poland; 18 GSK, Global Industrial Operations, Dresden, Germany; 19 Present Address: Pfizer VRD, Collegeville, PA USA; 20 GSK, Clinical Research and Development, King of Prussia, PA USA; 21 Present Address: Bill and Melinda Gates Foundation, Seattle, WA USA; 22 Present Address: PATH, Washington, DC USA

**Keywords:** Adults, Infants, Children, Quadrivalent, Influenza vaccine, Investigational, Manufacturing, Immunogenicity, Reactogenicity, Safety

## Abstract

**Background:**

GSK has modified the licensed monovalent bulk manufacturing process for its split-virion inactivated quadrivalent influenza vaccine (IIV4) to harmonize the process among different strains, resulting in an increased number of finished vaccine doses, while compensating for the change from inactivated trivalent influenza vaccine (IIV3) to IIV4. To confirm the manufacturing changes do not alter the profile of the vaccine, a clinical trial was conducted to compare IIV4 made by the currently licensed process with a vaccine made by the new (investigational) process (IIV4-I). The main objectives were to compare the reactogenicity and safety of IIV4-I versus IIV4 in all age groups, and to demonstrate the non-inferiority of the hemagglutination-inhibition (HI) antibody responses based on the geometric mean titer ratio of IIV4-I versus IIV4 in children.

**Methods:**

The Phase III, randomized, double-blind, multinational study included three cohorts: adults (18–49 years; *N* = 120), children (3–17 years; *N* = 821), and infants (6–35 months; *N* = 940). Eligible subjects in each cohort were randomized 1:1 to receive IIV4-I or IIV4. Both vaccines contained 15 μg of hemagglutinin antigen for each of the four seasonal virus strains. Adults and vaccine-primed children received one dose of vaccine, and vaccine-unprimed children received two doses of vaccine 28 days apart. All children aged ≥9 years were considered to be vaccine-primed and received one dose of vaccine.

**Results:**

The primary immunogenicity objective of the study was met in demonstrating immunogenic non-inferiority of IIV4-I versus IIV4 in children. The IIV4-I was immunogenic against all four vaccine strains in each age cohort. The reactogenicity and safety profile of IIV4-I was similar to IIV4 in each age cohort, and there was no increase in the relative risk of fever (≥38 °C) with IIV4-I versus IIV4 within the 7-day post-vaccination period in infants (1.06; 95% Confidence Interval: 0.75, 1.50; *p* = 0.786).

**Conclusions:**

The study demonstrated that in adults, children, and infants, the IIV4-I made using an investigational manufacturing process was immunogenic with a reactogenicity and safety profile that was similar to licensed IIV4. These results support that the investigational process used to manufacture IIV4-I is suitable to replace the current licensed process.

**Trial registration:**

ClinicalTrials.gov: NCT02207413; trial registration date: August 4, 2014.

**Electronic supplementary material:**

The online version of this article (10.1186/s12879-018-3079-8) contains supplementary material, which is available to authorized users.

## Background

Two phylogenetically and antigenically distinct influenza B lineages (B/Yamagata and B/Victoria) emerged globally in humans in the early 1980s, and have co-circulated in the US since 2001 [1]. Influenza B virus circulation is unpredictable, with both lineages co-circulating in recent years, meaning that trivalent influenza vaccines don’t offer optimal protection against influenza B [2, 3]. Consequently, some manufacturers have developed quadrivalent influenza vaccines to improve vaccine efficacy against influenza B, and in the 2012/13 influenza season, the World Health Organization (WHO) recommended four seasonal influenza viruses for seasonal quadrivalent influenza vaccines, including an influenza B/Yamagata and B/Victoria strain [4].

Quadrivalent influenza vaccines from various manufacturers were licensed based on studies demonstrating that quadrivalent versus trivalent vaccine have similar immunogenicity against common vaccine strains, but the quadrivalent vaccine had superior immunogenicity for the influenza B strain absent from the trivalent vaccine [5–21]. The studies also showed that quadrivalent and trivalent influenza vaccines have similar safety profiles despite the additional 15 μg of influenza B hemagglutinin antigen in the quadrivalent vaccine. To date, there has been only two efficacy study of a quadrivalent influenza vaccine, which was a randomized, observer-blinded assessment in children aged 6-35 months and 3–8 years of age [22, 23]. These studies showed that IIV4 versus control reduced PCR-confirmed ‘any influenza disease’ by 50 to 59.3% and reduced ‘moderate-to-severe influenza disease’ by 63 to 74.2% [22, 23].

GSK received marketing authorization in the United States in 2012 and in several European countries in 2013 for its inactivated quadrivalent influenza vaccine (IIV4) produced in Dresden, Germany [24–27]. GSK has introduced modifications to its licensed monovalent bulk manufacturing process for its IIV4 vaccine to harmonise process conditions for different influenza strains and to improve the manufacturing procedure to increase the number of finished vaccine doses, while compensating for the change from inactivated trivalent influenza vaccine (IIV3) to IIV4. The specification of the final product remained unchanged. While each of the modifications in itself was assessed as minor, the combined impact of the changes on the vaccine was difficult to assess using analytical and process data alone. Therefore the impact of the modifications on the benefit-risk profile of the vaccine was evaluated in a clinical study, assessing the immunogenicity and safety of IIV4-I and IIV4 in adults, children, and infants.

## Methods

### Design and participants

The Phase III, randomized, double-blind, multinational study was conducted to assess the safety and immunogenicity of IIV4 manufactured by an investigational process or by a licensed process in a cohort of adults (18–49 years), a cohort of children (3–17 years), and a cohort of infants (6–35 months). The study was conducted during the 2014/15 influenza season in Bangladesh, Czech Republic, France, Germany, Poland, Spain and the United States (ClinicalTrials.gov: NCT02207413; trial registration date: August 4, 2014).

To be eligible, participants in each age group had to be in stable health. Subjects were excluded if they had received 1) any non-registered drug or vaccine within 30 days or 2) any investigational or approved influenza vaccine (seasonal or pandemic) within 6 months of the first visit. Subjects were also excluded if they 3) were planning to participate in another clinical trial during the study, 4) were pregnant or breast-feeding, 5) were immunocompromised or receiving long-acting immune-modifying medicines, 6) had received immunoglobulins or any blood products within 3 months, or if they 7) had a history of allergy to any of the vaccine components or a history of Guillain-Barré Syndrome.

Adult participants were assessed from study entry until 21 days after vaccination. Children and infants were assessed from study entry until 28 days after the final vaccination. Cohorts were enrolled sequentially to limit risk to children and infants. Routine childhood vaccinations were permitted.

All adults or a parent/guardian of children provided written informed consent for participation, and children were required to assent if capable. The study was conducted in accordance with the Good Clinical Practice guidelines, the Declaration of Helsinki and applicable local regulations. All study documents were approved by the appropriate Institutional Review Boards.

The study design was reviewed and approved by The Paul-Ehrlich Federal Institute for Vaccines and Biomedicines (Langen, Germany) and the United States Food and Drug Administration Center for Biologics Evaluation and Research (CBER).

### Objectives

In adults (18–49 years) who received one dose of IIV4-I or IIV4, the primary objectives were: 1) to describe solicited injection-site adverse events (AEs) and general AEs within 7 days post-vaccination, including solicited oculorespiratory syndrome (ORS)-like symptoms within 3 days post-vaccination; 2) to describe unsolicited AEs within 21 days post-vaccination and serious adverse events (SAEs) and medically-attended adverse events (MAEs) during the entire study period. Secondary objectives included a description of hemagglutination-inhibition (HI) antibody titers for IIV4-I and IIV4 at baseline and 21 days after vaccination.

In children (3–17 years) who received one or two doses of IIV4-I or IIV4 the primary objectives were: 1) to demonstrate the immunogenic non-inferiority of IIV4-I compared with IIV4 for each vaccine strain based on the GMT ratio measured by HI assay 28 days after completion of the vaccination series, 2) to describe solicited injection site AEs and general AEs within 7 days post-vaccination, including solicited ORS-like symptoms within 3 days post-vaccination, 3) to describe unsolicited AEs within 28 days after vaccination and SAEs and MAEs during the entire study period. Secondary objectives included the assessment of the relative risk (RR) of myalgia within 7 days post-vaccination for IIV4-I versus IIV4 in children aged 5–17 years, and the assessment of baseline and post-vaccination HI antibody titers for IIV4-I and IIV4.

In infants (6–35 months) who received one or two doses of IIV4-I or IIV4 the primary objectives were to demonstrate sequentially: 1) the immunogenic non-inferiority of IIV4-I compared with IIV4 for each vaccine strain based on geometric mean titer (GMT) ratio measured by HI assay 28 days after completion of the vaccination series; 2) no significant increase in subjects who reported fever ≥38 °C with IIV4-I compared with IIV4 within the 7-day post-vaccination periods after dose 1 or dose 2. Secondary objectives included the assessment of the RR of fever ≥38 °C in the IIV4-I group compared with the IIV4 group within 7 days post-vaccination after dose 1 and dose 2. Further secondary assessments were solicited injection site AEs and general AEs within 7 days post-vaccination, including solicited ORS-like symptoms within 3 days post-vaccination, unsolicited AEs (28 days), SAEs, and MAEs (entire study period), and baseline and post-vaccination HI antibody titers for IIV4-I and IIV4.

A further secondary objective in a group pooled from the infant and children cohorts including subjects aged 6 months–4 years was to describe the RR of fever ≥38 °C and >39 °C within 2 days after dose 1 or dose 2 in the IIV4-I group compared with the IIV4 group.

### Enrolment and randomization

The adults, children, and infants cohorts were enrolled sequentially. Adults were enrolled and vaccinated first, and a safety review was conducted by the sponsor’s internal Safety Review Committee (iSRC) to review the 7 days post-vaccination safety data. Upon the receipt of iSRC’s endorsement based on the safety review in adults, enrolment of children aged 3–17 years began, and enrolment of infants aged 6–35 months started after the first 100 subjects in the 3–17 years cohort were vaccinated and no life-threatening related SAE(s) were reported within 2 days after dose 1.

The randomization list and allocation of vaccines was generated using a software program developed for use in SAS (Cary, NC, USA) by GSK, Rixensart, Belgium. Vaccine allocation of the subjects at each study center was performed by investigators using an internet-based central randomization system. Eligible subjects in each age cohort were randomized 1:1 to receive IIV4-I or IIV4. The randomization algorithm used a minimization procedure in each age cohort: randomization in adults accounted for study center, age (18–33 years and 34–49 years), and influenza vaccination history in the previous season; randomization in children accounted for study center, age (3–4 years, 5–8 years, and 9–17 years), vaccine-priming status in children aged < 9 years, and influenza vaccination history in the previous season in children aged ≥9 years; and randomization in infants accounted for study center, age (6–17 months and 18–35 months), and vaccine-priming status.

### Vaccines and vaccination

The IIV4-I and the IIV4 (*Influsplit Tetra, Fluarix Tetra, Fluarix Quadrivalent*) were developed and manufactured by GSK in Dresden, Germany. IIV4-I and IIV4 were prepared from influenza viruses grown in embryonated chicken eggs. Virus is disrupted in a solution containing detergent and inactivated by the consecutive effect of sodium deoxycholate and formaldehyde. The split virus is suspended in sodium phosphate-buffered isotonic sodium chloride solution. IIV4-I was manufactured by a bulk manufacturing process in which conditions for different influenza strains were harmonised, such as composition of process media or use of excipients. The changes did not affect the conditions of virus replication, or inactivation. The specification of the final vaccine remains the same as for IIV4.

Each 0.5 mL dose of vaccine contained 15 μg of hemagglutinin antigen for each of the influenza strains recommended by the WHO for the 2014/15 influenza season in the Northern Hemisphere: A/H1N1 (A/Christchurch/16/2010, an A/California/7/2009 like stain), A/H3N2 (A/Texas/50/2012, antigenically like the cell propagated A/Victoria/361/2011 strain), B/Yamagata lineage (B/Massachusetts/2/2012), and B/Victoria lineage (B/Brisbane/60/2008) [28].

Adults received one dose of vaccine (Day 0), infants and children aged 6 months–8 years who were vaccine-primed received one dose of vaccine (Day 0) and those who were vaccine-unprimed received two doses of vaccine 28 days apart (Day 0 and Day 28). All children aged ≥9 years were considered to be vaccine-primed and received one dose of vaccine. Vaccines were administered by blinded personnel in the deltoid of the non-dominant arm (adults and children ≥12 months), or in the antero-lateral thigh (infants aged <12 months).

Children were defined as vaccine-primed if they had received ≥1 dose(s) of seasonal influenza vaccine in the previous influenza season (2013/14), or ≥2 doses of seasonal influenza vaccine since 1st July 2010, or ≥2 doses of seasonal influenza vaccine before 1st July 2010 and ≥1 dose(s) of A(H1N1)pdm09 vaccine, or ≥ 1 dose(s) of seasonal influenza vaccine before 1st July 2010 and ≥1 dose(s) of seasonal influenza vaccine since 1st July 2010. The definition of vaccine-priming status was based on US Advisory Committee on Immunization Practices recommendations [29].

### Assessments

#### Immunogenicity

Immunogenicity was assessed at Day 0 and Day 28 (infants and children) or Day 21 (adults) in subjects who received one dose of vaccine, and at Day 0 and Day 56 in infants and children who received two doses of vaccine. Immune responses to each vaccine strain were assessed by HI assay using an established method [30].

HI antibody responses were described as the arithmetic mean of the log-10 transformed inverse geometric mean titres (GMT), seroprotection rate (SPR; proportion with post-vaccination titer ≥1:40), and the seroconversion rate (SCR; proportion with antibody titer < 1:10 at baseline and with post-vaccination titer of ≥1:40, or pre-vaccination titer of ≥1:10 and a ≥4-fold post-vaccination increase in titer). Subjects with HI antibody titers of ≥1:10 were considered to be seropositive.

#### Solicited AEs

The incidence and severity of injection site AEs and general AEs was recorded by adults and by parents/guardians on diary cards during 7 days after receipt of each dose of vaccine. Injection site symptoms were: pain, swelling, and redness; general symptoms in children aged ≥5 years and adults were fatigue, gastrointestinal (GI) symptoms, headache, joint pain, myalgia, shivering, and fever. General symptoms in infants and children aged <5 years were drowsiness, irritability/fussiness, loss of appetite, and fever. Solicited ORS-like symptoms were: bilateral red eyes, chest tightness, cough, difficulty breathing, wheezing, hoarseness, sore throat, swallowing difficulty and swelling of the face.

#### Unsolicited AEs

In the adult cohort, unsolicited AEs were assessed within 21 days post-vaccination, and in the infant and children cohorts, unsolicited AEs were assessed within 28 days after the receipt of vaccine. MAEs and SAEs were assessed during the whole study period, including the allowed visit intervals of up to 23 days for adults, and up to 42 days post-vaccination for children.

#### Severity and causality of AEs

Solicited and unsolicited AEs were graded for severity: Grade 1 symptoms were those which caused mild discomfort; Grade 2 symptoms were those which caused moderate discomfort but did not prevent normal activities; Grade 3 symptoms were those which caused severe discomfort which prevented normal activities. Fever was defined as an axillary temperature of ≥38.0 °C and Grade 3 fever as >39.0 °C. All injection-site AEs were deemed to be vaccine-related and investigators provided causality assessments for solicited general AEs and unsolicited AEs.

### Statistical analyses

The target sample size of adults (18–49 years) was 120, which was based on an evaluable population of 100 participants with a 15% drop-out rate. This sample size was considered to be an adequate sample for the pilot safety assessment in adults before the sequential enrolment of children.

The target sample size in the children (3–17 years) cohort was based on the power to detect immunogenic non-inferiority for IIV4-I compared with IIV4 of the HI GMT ratio for 4 vaccine strains 28 days after the completion of the vaccination series. The target population in children was 360 evaluable subjects in each vaccine group, which would detect immunogenic non-inferiority with 98% global power using a 1-sided, 2-sample t-test and to meet the upper limit (UL) of the 95% confidence interval (CI) of the GMT ratio was ≤1.5. The target sample size in the infant (6–35 months) cohort was based on 420 evaluable subjects needed in each group to detect non-inferiority of HI GMT ratio for IIV4-I compared with IIV4 with 92% global power using a 1-sided, 2-sample t-test to meet the UL of the 95% CI of the GMT ratio ≤1.5. A total of 470 vaccinated infants provides 90% power to rule out the relative risk of fever (38.0°C) >2 for IIV4-I/IIV4 using a 1-sided Score test [31].

GMT ratios were derived using the Analysis of Covariance (ANCOVA) on the log10 transformed titers adjusted by age and pre-vaccination titer. Immunogenicity analyses of GMT, SPR, and SCR were described as point estimates with 95% CIs. All immunogenicity analyses were performed on the per-protocol immunogenicity cohort for each age group including eligible subjects without protocol deviation who had serological data available at a given time point.

Solicited and unsolicited AEs were described as percentages with 95% CIs. All safety assessments were performed in the total vaccinated cohort (TVC) including all subjects who received at least 1 dose of vaccine. The relative risk (RR) of myalgia and fever was calculated with a 95% CIs.

## Results

**Fig. 1:**
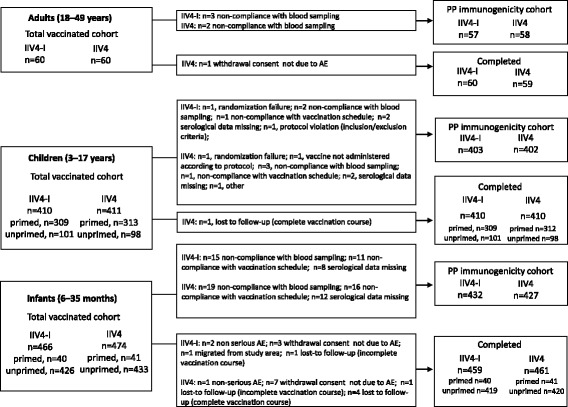
Subject disposition. IIV4-I, quadrivalent inactivated influenza vaccine manufacturing by investigational process; IIV4, licensed quadrivalent inactivated influenza vaccine; AE, adverse event; SAE, serious adverse event; PP, per-protocol

Enrolment started on 18 August 2014 and the last study visit was completed on 18 April 2015. The TVC of adults (aged 18–49 years) included 120 participants of which 119 completed the final study visit; the TVC of children (aged 3–17 years) included 821 participants of which one was lost to follow-up; the TVC of infants (aged 6–35 months) included 940 participants of which 920 completed the final study visit (Fig. 1). The baseline demographic characteristics in each age cohort are shown in Table 1. The vaccine groups were generally well balanced regarding age, geographic ancestry and gender.

**Table 1:** Baseline demographics in the total vaccinated cohort

	Infants		Children		Adults	
	6–35 months		3–17 years		18–49 years	
	IIV4-I *N* = 466	IIV4 *N* = 474	IIV4-I *N* = 410	IIV4 *N* = 411	IIV4-I *N* = 60	IIV4 *N* = 60
Mean age, (SD)	19.7 (8.0) months	19.9 (8.3) months	9.4 (4.2) years	9.4 (4.2) years	29.8 (8.7) years	31.2 (9.3) years
Median age, (range)	19 (4–36) months	19 (5–35) months	9.0 (3–18) years	10.0 (3–18) years	28.0 (19–49) years	29.0 (18–49) years
Male, n (%)	243 (52.1)	265 (55.9)	214 (52.1)	224 (54.5)	19 (31.7)	25 (41.7)
White - Caucasian/European Heritage, n (%)	329 (70.6)	337 (71.1)	378 (92.2)	370 (90.0)	60 (100)	56 (93.3)
African Heritage/African American, n (%)	10(2.1)	10 (2.1)	11 (2.7)	17 (4.1)	0	2 (3.3)
Asian - South East Asian Heritage, n (%)	91 (19.5)	91 (19.2)	0	0	0	2 (3.3)
White - Arabic/North African Heritage, n (%)	28 (6.0)	23 (4.9)	13 (3.2)	15 (3.6)	0	0

*IIV4-I* quadrivalent inactivated influenza vaccine manufacturing by investigational process, *IIV4* licensed quadrivalent inactivated influenza vaccine, *SD* standard deviation, *N* number of subjects with ≥1 vaccine dose, *n* number of subjects fulfilling the demographic

In children aged 3–17 years, in the IIV4-I and IIV4 groups, 309 and 313 children respectively were primed, and 101 and 98 children, respectively were unprimed (Fig. 1). In infants aged 6–35 months, in the IIV4-I and IIV4 groups, 40 and 41 children respectively were primed, and 426 and 433 infants, respectively were unprimed.

### Immunogenicity

#### Adults

**Fig. 2:**
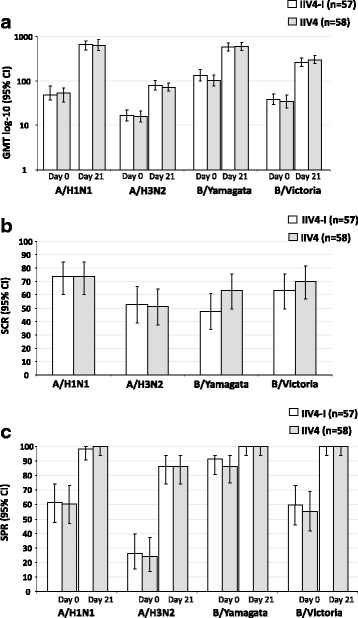
Hemagglutinin-inhibition antibody responses in adults aged 18–49 years in the per-protocol immunogenicity cohort. **a** = GMT, geometric mean titer; **b** = SCR, seroconversion rate; **d**= SPR, seroprotection rate; IIV4-I, quadrivalent inactivated influenza vaccine manufacturing by investigational process; IIV4, licensed quadrivalent inactivated influenza vaccine; CI, confidence interval; n, number of subjects in the per-protocol cohort

The per-protocol immunogenicity population included 57 adults (18–49 years) in the IIV4-I group and 58 in the IIV4 group. Both vaccines were immunogenic, and 95% CIs for the SCRs and SPRs overlapped between the IIV4-I and IIV4 groups. Against each vaccine strain 21 days after vaccination: the SCRs varied from 47.4 to 73.7% and from 50.9 to 73.7% for IIV4-I and IIV4 groups, respectively (Fig. 2).

#### Children

The per-protocol immunogenicity population included 403 children (3–17 years) in the IIV4-I group and 402 in the IIV4 group. Immunogenic non-inferiority in terms of the GMT ratio for IIV4-I compared with IIV4 was demonstrated for all four vaccine strains (UL 95% CI ≤1.5) (Table 2). Both vaccines were immunogenic against each vaccine strain 28 days after vaccination (Table 3). Against each vaccine strain, the SCRs varied from 47.6 to 70.7% and from 45.5 to 71.4% for IIV4-I and IIV4 groups, respectively.

**Table 2:** Immunogenic non-inferiority 28 days after last vaccination for IIV4 versus IIV4-I in children and infants in the per-protocol immunogenicity cohort

	IIV4-I	IIV4	IIV4/IIV4-I
	Adjusted GMT	Adjusted GMT	Adjusted GMT ratio (95% CI)
Infants, 6–35 months	*N* = 431	*N* = 423^a^	
A/H1N1	98.0	105.3	1.07 (0.90, 1.28)
A/H3N2	47.7	56.3	1.18 (1.00, 1.39)
B/Yamagata	99.2	106.4	1.07 (0.91, 1.27)
B/Victoria	32.2	37.7	1.17 (0.99, 1.38)
Children, 3–17 years	*N* = 403	*N* = 402	
A/H1N1	707.3	684.9	0.97 (0.85, 1.11)
A/H3N2	160.6	168.8	1.05 (0.94, 1.18)
B/Yamagata	496.0	509.4	1.03 (0.91, 1.16)
B/Victoria	240.8	250.4	1.04 (0.90, 1.21)

*IIV4-I* quadrivalent inactivated influenza vaccine manufacturing by investigational process, *IIV4* licensed quadrivalent inactivated influenza vaccine, *GMT* geometric mean titer adjusted for age and pre-vaccination titers, *CI* confidence interval

^a^A/H1N1, *N* = 424; N, number of subjects in the per-protocol cohort with both pre-and post-vaccination results available

**Table 3:** Hemagglutinin-inhibition antibody titers in children and infants in the per-protocol immunogenicity cohort

Strain & cohort		Days	Seropositive		GMT		SPR		SCR	
			n/*N*; % (95% CI)		value (95% CI)		n/*N*; % (95% CI)		n/*N*; % (95% CI)	
			IIV4-I	IIV4	IIV4-I	IIV4	IIV4-I	IIV4	IIV4-I	IIV4
A/H1N1	Children	0	354/403; 87.8 (84.2, 90.9)	364/402; 90.5 (87.3, 93.2)	80.2 (69.2, 93.0)	87.7 (76.1, 101.0)	308/403; 76.4 (72.0, 80.5)	314/402; 78.1 (73.7, 82.1)	–	–
		56 or 28^a^	402/403; 99.8 (98.6, 100)	401/402; 99.8 (98.6, 100)	698.0 (629.6, 773.9)	694.1 (625.8, 769.7)	393/403; 97.5 (95.5, 98.8)	395/402; 98.3 (96.4, 99.3)	274/403; 68.0 (63.2, 72.5)	269/402; 66.9 (62.1, 71.5)
	Infants	0	109/431; 25.3 (21.3, 29.7)	111/424; 26.2 (22.1, 30.6)	11.1 (9.6, 12.8)	11.2 (9.7, 12.9)	84/431; 19.5 (15.9, 23.5)	83/424; 19.6 (15.9, 23.7)	–	–
		56 or 28^a^	406/432; 94.0 (91.3, 96.0)	408/427; 95.6 (93.1, 97.3)	97.5 (82.1, 115.7)	105.5 (88.2, 126.1)	303/432; 70.1 (65.6, 74.4)	289/427; 67.7 (63.0, 72.1)	287/431; 66.6 (61.9, 71.0)	275/424; 64.9 (60.1, 69.4)
A/H3N2	Children	0	346/403; 85.9 (82.1, 89.1)	345/402; 85.8 (82.0, 89.1)	38.9 (34.6, 43.8)	41.9 (37.1, 47.3)	245/403; 60.8 (55.8, 65.6)	252/402; 62.7 (57.8, 67.4)	–	–
		56 or 28^a^	400/403; 99.3 (97.8, 99.8)	401/402; 99.8 (98.6, 100)	158.2 (143.7, 174.2)	171.4 (156.3, 188.0)	377/403; 93.5 (90.7, 95.7)	378/402; 94.0 (91.2, 96.1)	192/403; 47.6 (42.7, 52.6)	183/402; 45.5 (40.6, 50.5)
	Infants	0	79/431; 18.3 (14.8, 22.3)	91/423; 21.5 (17.7, 25.7)	7.5 (6.8, 8.2)	8.4 (7.5, 9.3)	55/431; 12.8 (9.8, 16.3)	67/423; 15.8 (12.5, 19.7)	–	–
		56 or 28^a^	388/432; 89.8 (86.6, 92.5)	403/427; 94.4 (91.8, 96.4)	45.2 (39.0, 52.3)	59.9 (51.7, 69.4)	232/432; 53.7 (48.9, 58.5)	259/427; 60.7 (55.8, 65.3)	217/431; 50.3 (45.5, 55.2)	236/423; 55.8 (50.9, 60.6)
B/Yamagata	Children	0	325/403; 80.6 (76.4, 84.4)	333/402; 82.8 (78.8, 86.4)	58.1 (49.7, 68.0)	70.8 (60.4, 83.0)	266/403; 66.0 (61.2, 70.6)	281/402; 69.9 (65.2, 74.3)	–	–
		56 or 28^a^	403/403; 100 (99.1, 100)	401/402; 99.8 (98.6, 100)	479.0 (434.1, 528.5)	527.6 (475.5, 585.3)	396/403; 98.3 (96.5, 99.3)	395/402; 98.3 (96.4, 99.3)	273/403; 67.7 (62.9, 72.3)	268/402; 66.7 (61.8, 71.3)
	Infants	0	105/431; 24.4 (20.4, 28.7)	99/423; 23.4 (19.4, 27.7)	8.3 (7.5, 9.1)	7.9 (7.2, 8.6)	53/431; 12.3 (9.3, 15.8)	49/423; 11.6 (8.7, 15.0)	–	–
		56 or 28^a^	415/432; 96.1 (93.8, 97.7)	409/427; 95.8 (93.4, 97.5)	100.8 (87.8, 115.8)	105.4 (91.8, 121.0)	329/432; 76.2 (71.9, 80.1)	331/427; 77.5 (73.3, 81.4)	318/431; 73.8 (69.4, 77.9)	321/423; 75.9 (71.5, 79.9)
B/Victoria	Children	0	285/403; 70.7 (66.0, 75.1)	287/402; 71.4 (66.7, 75.8)	27.3 (23.8, 31.3)	28.8 (25.0, 33.1)	192/403; 47.6 (42.7, 52.6)	195/402; 48.5 (43.5, 53.5)	–	–
		56 or 28^a^	398/403; 98.8 (97.1, 99.6)	394/402; 98.0 (96.1, 99.1)	237.6 (210.4, 268.3)	253.7 (222.7, 289.1)	375/403; 93.1 (90.1, 95.3)	374/402; 93.0 (90.1, 95.3)	285/403; 70.7 (66.0, 75.1)	287/402; 71.4 (66.7, 75.8)
	Infants	0	30/431; 7.0 (4.7, 9.8)	30/423; 7.1 (4.8, 10.0)	5.7 (5.4, 6.1)	5.7 (5.4, 6.1)	17/431; 3.9 (2.3, 6.2)	16/423; 3.8 (2.2, 6.1)	–	–
		56 or 28^a^	359/432; 83.1 (79.2, 86.5)	366/427; 85.7 (82.0, 88.9)	32.1 (28.1, 36.7)	38.0 (33.2, 43.5)	214/432; 49.5 (44.7, 54.4)	217/427; 50.8 (46.0, 55.7)	213/431; 49.4 (44.6, 54.2)	211/423; 49.9 (45.0, 54.8)

*Children*, 3–17 years; *infants*, 6–35 months; *IIV4-I*, quadrivalent inactivated influenza vaccine manufacturing by investigational process; *IIV4*, licensed quadrivalent inactivated influenza vaccine; *CI*, confidence interval; *GMT*, geometric mean titer; *SCR*, seroconversion rate; *SPR*, seroprotection rate

*n*, number of subjects fulfilling immunogenicity definition; *N*, number of subjects in the per-protocol cohort with results available

^a^28 days after final vaccination, i.e. Day 28 in primed subjects or Day 56 in unprimed subjects

#### Infants

The per-protocol immunogenicity population included 432 infants (6–35 months) in the IIV4-I group and 427 in the IIV4 group. Immunogenic non-inferiority in terms of GMT ratio for IIV4-I compared with IIV4 was demonstrated for all four vaccine strains (UL 95% CI ≤1.5) (Table 2). Both vaccines were immunogenic against each vaccine strain 28 days after vaccination (Table 3). Against each vaccine strain, the SCRs varied from 49.4 to 73.8% and from 49.9 to 75.9% for IIV4-I and IIV4 groups, respectively.

### Reactogenicity and safety

#### Adults (18–49 years)

The TVC of adults included 60 in both IIV4-I and IIV4 groups. Overall, the reactogenicity profiles were consistent between the IIV4-I and IIV4 vaccine groups.

**Fig. 3:**
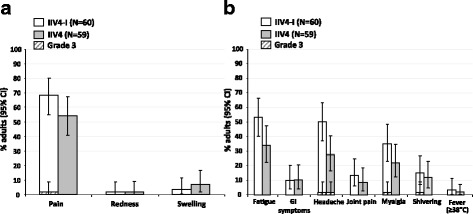
Solicited injection-site adverse events (**a**) and general adverse events (**b**) in adults aged 18–49 years in the total vaccinated cohort. IIV4-I, quadrivalent inactivated influenza vaccine manufacturing by investigational process; IIV4, licenced quadrivalent inactivated influenza vaccine; CI, confidence interval; GI, gastrointestinal; *N*, number of subjects in the in the total vaccinated cohort who returned diary cards

Within the 7 days post-vaccination, pain was the most frequent solicited injection site AE in the IIV4-I group (41/60; 68.3%) and the IIV4 group (32/59; 54.2%), of which most reports were Grade 1 or 2 (Fig. 3). There was 1 report of Grade 3 pain in the IIV4-I group. Swelling was reported by 3.3% (2/60) and 6.8% (4/59) of the IIV4-I and IIV4 groups, respectively, and redness was reported by 1 adult in each vaccine group. Headache and fatigue were the most common solicited general AEs in the IIV4-I group (30/60; 50.0% and 32/60; 53.3%, respectively) and in the IIV4 group (16/59; 27.1% and 20/59; 33.9%, respectively).

Solicited ORS-like symptoms during 3 days post-vaccination were uncommon in both groups (Additional file 1**)**.

During 21 days post-vaccination, ≥1 unsolicited AE was reported by 23.3% (14/60) of the IIV4-I and IIV4 groups, of which 3 (5.0%) and 2 (3.3%) reports, respectively, were Grade 3 in severity (Table 4). During the entire study period, there was 1 SAE in each group (each 1.7%), and there were 9 (15.0%) and 8 (13.3%) MAEs in the IIV4-I and IIV4 groups, respectively (Additional file 2 and Additional file 3). None of the SAEs or MAEs was considered by the investigator to be causally-related to vaccination.

**Table 4:** Global summary of unsolicited adverse events in adults, children, and infants in the total vaccinated cohort

	Infants		Children		Adults	
	6–35 months		3–17 years		18–49 years	
	IIV4-I *N* = 466	IIV4 *N* = 474	IIV4-I *N* = 410	IIV4 *N* = 411	IIV4-I *N* = 60	IIV4 *N* = 60
Unsolicited AEs for 21 days (adults) or 28 days (infants & children) post-last vaccination						
≥1 AE, n (%)	243 (52.1)	262 (55.3)	83 (20.2)	86 (20.9)	14 (23.3)	14 (23.3)
≥1 Grade 3 AE, n (%)	33 (7.1)	31 (6.5)	12 (2.9)	8 (1.9)	3 (5.0)	2 (3.3)
≥1 AE causally-related to vaccination, n (%)	6 (1.3)	3 (0.6)	10 (2.4)	7 (1.7)	2 (3.3)	1(1.7)
≥1 Grade 3 AE causally-related to vaccination, n (%)	1 (0.2) [bronchitis]	0	1 (0.2) [axillary pain]	1 (0.2) [injection site pustule]	0	0
MAEs for entire study^a^						
≥1 MAE, n (%)	235 (50.4)	252 (53.2)	59 (14.4)	52 (12.7)	9 (15.0)	8 (13.3)
≥1 Grade 3 MAE, n (%)	35 (7.5)	29 (6.1)	7 (1.7)	6 (1.5)	3 (5.0)	1 (1.7)
≥1 MAE causally-related to vaccination, n (%)	2 (0.4)	0	2 (0.5)	0	0	0
≥1 Grade 3 MAE causally-related to vaccination, n (%)	1 (0.2) [bronchitis]	0	0	0	0	0
SAEs for entire study^a^						
≥1 SAE, n (%)	7 (1.5)	11 (2.3)	1 (0.2)	0	1 (1.7)	1 (1.7)
≥1 SAE causally-related to vaccination, n (%)	0	0	0	0	0	0

*IIV4-I* quadrivalent inactivated influenza vaccine manufacturing by investigational process, *IIV4* licensed quadrivalent inactivated influenza vaccine, *AE* adverse event; *MAE*, medically-attended adverse event, *SAE* serious adverse event

*N*, number of subjects with ≥1 vaccine dose; *n*, number of subjects reporting the event

^a^including the allowed visit interval of up to 23 days post-vaccination for the adults and up to 42 days post-last vaccination for the children

#### Children (3–17 years)

The TVC of children included 410 in the IIV4-I group and 411 in the IIV4 group. The reactogenicity profiles of IIV4-I and IIV4 were similar.

**Fig. 4:**
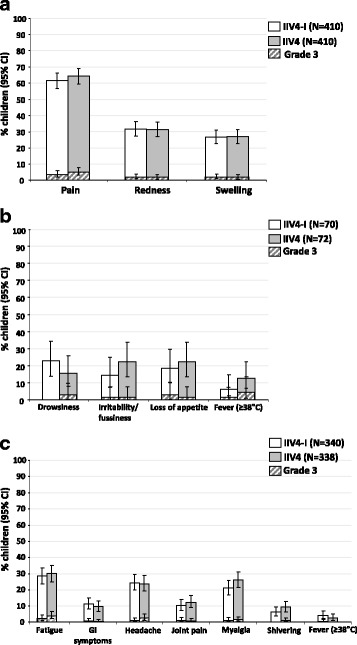
Solicited injection-site events in children aged 3–17 years (**a**) and general adverse events in children aged 3–4 years (**b**) and children aged 5–17 years (**c**) in the total vaccinated cohort. IIV4-I, quadrivalent inactivated influenza vaccine manufacturing by investigational process; IIV4, licenced quadrivalent inactivated influenza vaccine; CI, confidence interval; GI, gastrointestinal; *N*, number of subjects in the total vaccinated cohort who returned diary cards the per-protocol cohort

During 7-days post-vaccination overall, pain was the most frequent solicited injection site AE in the IIV4-I (252/410; 61.5%) and the IIV4 (264/410; 64.4%) groups, of which 14 (3.4%) and 21 (5.1%), respectively, were Grade 3 in severity (Fig. 4). Redness and swelling were reported by 129 (31.5%) and 109 (26.6%) children in the IIV4-I group, and 128 (31.2%) and 110 (26.8%) children in the IIV4 group.

Within 7 days post-vaccination overall, the most common solicited general AE in children aged 3–4 years was drowsiness in the IIV4-I group (16/70; 22.9%) and in the IIV4 group (11/72; 15.3%), of which 2 (2.8%) reports in the IIV4 group were Grade 3 in severity (Fig. 4). In children aged 3–4 years overall, there were 4 (5.7%) and 9 (12.5%) reports of fever ≥38.0 °C in the IIV4-I and IIV4 groups, respectively. Overall in children aged 5–17 years, fatigue was the most common solicited general AE in the IIV4-I group (97/340; 28.5%) and in the IIV4 group (101/338; 29.9%) (Fig. 4).

In the TVC, within 3 days post-vaccination the most common ORS-like symptoms were cough, red eyes, sore throat, and hoarseness (Additional file 1). In children aged 5–17 years, the RR for IIV4-I compared with IIV4 for myalgia within 7 days post-vaccination overall was 0.8 (95% CI: 0.58, 1.11; *p* = 0.1914).

In the TVC, 83 (20.2%) and 86 (20.9%) children in the IIV4-I and IIV4 groups, respectively, reported ≥1 AE during 28 days post-vaccination (Table 4). There were 12 (2.9%) and 8 (1.9%) children with Grade 3 AEs and 10 (2.4%) and 7 (1.7%) AEs, in the IIV4-I and IIV4 groups, respectively, which were considered by the investigator to be causally-related to vaccination.

During the entire study period, there were 59 (14.4%) and 52 (12.7%) MAEs (most commonly upper respiratory tract infection, bronchitis, pharyngitis, and nasopharyngitis) in the IIV4-I and IIV4 groups, respectively (Additional file 3). Only 1 SAE,viral meningitis was observed in the IIV4-I group but it was not considered by the investigator to be vaccine-related (Additional file 2).

#### Infants (6–35 months)

The TVC of infants included 466 and 474 in the IIV4-I and IIV4 groups, respectively. Within 7 days post-vaccination overall, there were 72 (15.6%) and 69 (14.7%) reports of fever ≥38 °C in the IIV4-I and IIV4 groups, respectively, and the RR for fever with IIV4-I versus IIV4 was 1.06 (0.75, 1.50; *p* = 0.7868). After dose 1, the RR of fever ≥38 °C for IIV4-I versus IIV4 was 0.94 (95% CI: 0.59, 1.50; *p* = 0.885) and 1.00 (95% CI: 0.63, 1.59; *p* = 1.000) after dose 2 (Table 5).

**Table 5:** Relative risk of fever ≥38.0 °C for IIV4-I versus IIV4, within 7 days post-vaccination, in infants 6–35 months in the total vaccinated cohort

Axillary temperature, °C		IIV4-I n/*N*; %	IIV4 n/*N*; %	IIV4-I/IIV4 RR (95% CI)	*p*-value
Dose 1	≥38.0	39/462; 8.4	42/ 470; 8.9	0.94 (0.59, 1.50)	0.8851
Dose 2	≥38.0	40/420; 9.5	40/421; 9.5	1.00 (0.63, 1.59)	1.000
Overall	≥38.0	72/462; 15.6	69/470;14.7	1.06 (0.75, 1.50)	0.7868

*IIV4-I* quadrivalent inactivated influenza vaccine manufacturing by investigational process, *IIV4* licensed quadrivalent inactivated influenza vaccine, *RR* relative risk, *N*, number of subjects with ≥1 vaccine dose and returned diary cards; *n*, number of subjects reporting the event

**Fig. 5:**
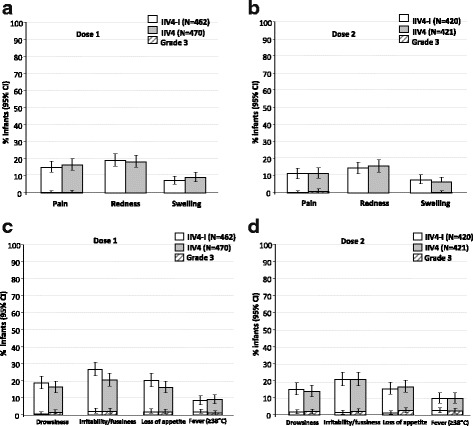
Solicited injection-site adverse events after the first (**a**) and second (**b**) dose, and general adverse events after the first (**c**) and second dose (**d**) in infants aged 6–35 months in the total vaccinated cohort. IIV4-I, quadrivalent inactivated influenza vaccine manufacturing by investigational process; IIV4, licenced quadrivalent inactivated influenza vaccine; CI, confidence interval; N, number of subjects in the total vaccinated cohort who returned diary cards

The most common solicited injection site AE within 7 days post-vaccination after dose 1 and dose 2 was pain in the IIV4-I group (69/462; 14.9%, and 47/420; 11.2%, respectively) and IIV4 group (77/470; 16.4%, and 48/421; 11.4%, respectively) (Fig. 5). After dose 1, redness and swelling were reported by 88 (19.0%) and 33 (7.1%) of the IIV4-I group, and 86 (18.3%) and 42 (8.9%) of the IIV4 group. After dose 2, redness and swelling were reported by 61 (14.5%), and 32 (7.6%) of the IIV4-I group, and 66 (15.7%) and 27 (6.4%) of the IIV4 group,. Grade 3 local events were uncommon. After dose 1 and 2, the most common solicited general AE was irritability/fussiness in the IIV4-I group (124/462; 26.8% and 87/420; 20.7%), and in the IIV4 group (96/470; 20.4% and 87/421; 20.7%). The most common solicited symptoms of ORS-like within 3 days post-vaccination overall was cough in the IIV4-I group (73/462; 15.8%) and in the IIV4 group (85/470; 18.1%) (Additional file 1).

During 28 days post-vaccination, 243 (52.1%) and 262 (55.3%) of participants in the IIV4-I and IIV4 groups reported ≥1 unsolicited AE, of which 33 (7.1%) and 31 (6.5%), were Grade 3 in severity (Table 4).

During the entire study period, there were 235 (50.4%) and 252 (53.2%) MAEs in the IIV4-I and IIV4 groups, which were most commonly upper respiratory tract infection, bronchitis, and gastroenteritis (Additional file 3). There were 7 (1.5%) and 11 (2.3%) SAEs in the IIV4-I and IIV4 groups none of which were considered by the investigator to be vaccine-related (Additional file 2). There was a report of febrile seizure for 1 infant in the IIV4 group which occurred 31 days after study vaccination and concomitantly with an adenovirus infection that was not considered by the investigator to be causally-related to vaccination.

#### Children aged < 5 years

Cases of fever ≥38.0 °C within 2 days post-vaccination in children aged <5 years were reported in 5.3% (*n* = 28) of the IIV4-I group and 5.7% (*n* = 31) of the IIV4 group resulting in a RR (95% CI) of 0.92 (0.53, 1.59; *p* = 0.8507). There were 4 and 1 reports of fever >39.0 °C within 2 days post-vaccination in the IIV4-I and IIV4 groups, respectively resulting in a RR for IIV4-I versus IIV4 of 4.08 (0.40, 200.69; *p* = 0.3635).

## Discussion

The need to test the impact of manufacturing changes in clinical trials should be based on a careful risk assessment of the changes to the product and a detailed evaluation of the comparability of critical quality attributes. Changes to the inactivation process or other critical process steps, or changes that require modification of the product-release specifications are more likely to require clinical evaluation than other changes that do not affect critical process steps, critical quality attributes or specification limits. This Phase III, double-blind, multinational study was conducted to assess the immunogenicity and safety of a inactivated quadrivalent influenza vaccine manufactured by an investigational process (IIV4-I) to ensure it is suitable to replace the vaccine manufactured by the current licensed process (IIV4).

The study showed that the reactogenicity and safety profile of IIV4-I and IIV4 were similar in each age cohort. The immunogenicity analyses showed that antibody titers against each vaccine strain fulfilled the definition of non-inferiority for IIV4-I versus IIV4 in infants and in children, and that GMTs, SCRs, and SPRs were similar between vaccine groups in each age cohort. In adults, both vaccines were immunogenic against all vaccine strains, although SCRs against H3N2 were lower than the other strains in both vaccine groups.

Influenza vaccines are only moderately immunogenic in infants and young children who have had limited previous exposure to vaccines and viruses such that two doses of vaccine are recommended in influenza vaccine-naïve children aged ≤9 years to achieve antibody titers that are considered protective [32]. There are no immunogenic thresholds specified by regulatory authorities as a surrogate of protection against influenza in children. As well as demonstrating immunogenic non-inferiority at 28 days after the vaccination series, we observed robust antibody responses in infants and children. In children who received IIV4-I, SCRs were 47.6–70.7%, and in infants who received IIV4-I, SCRs were 49.4–73.8%*.*

The safety and reactogenicity evaluations used conventional endpoints but focused on the incidence of fever and of signs and symptoms of ORS because both outcomes have been associated with manufacturing process changes in the past [33–36]. For example, the increased prevalence of febrile convulsions in children aged <5 years in Australia and New Zealand who received *Fluvax* (CSL Biotherapies) in 2010 demonstrated the potential for alterations in the safety profile of vaccines from season to season and between vaccine brands [34, 35]. The increased pyrogenicity for *Fluvax* versus other IIV3s was thought to be associated with the different manufacturing processes used by CSL Biotherapies than in the other IIV3s [34, 35, 37]. ORS was first described as an influenza vaccine-associated AE in Canada during the 2000/01 influenza vaccination campaign, and was linked to the domestically-produced IIV3 which contained unsplit and aggregated influenza virions at higher than expected levels [33, 36]. The symptoms of ORS usually present within 24 h of vaccination and resolve within 72 h of onset, and can include bilateral red eyes, facial oedema, and respiratory symptoms such as sore throat, cough, and wheezing. In our study, in the 6–35 months cohort, fever ≥38 °C within 7 days post-vaccination was selected as an objective comparative measure of reactogenicity between the vaccines. In the infant population overall, the incidence of fever ≥38 °C was 15.6% and 14.7% in the IIV4-I and IIV4 groups, respectively, and the RR for post-vaccination fever ≥38 °C for IIV4-I/IIV4 was 1.06 (95% CI: 0.75, 1.50). In children aged 6 months-<5 years, within 2 days post-vaccination there were 4 (0.8%) reports and 1 (0.2%) report of Grade 3 fever in the IIV-I and IIV4 groups, respectively; the observed RR (4.08) was due to the very low incidence. There were no reports of febrile convulsion considered by the investigator to be causally-related to vaccination. Within 3 days after vaccination, cough was the most reported ORS-like symptom in the IIV4-I and IIV4 groups in infants (15.8% and 18.1%, respectively) and children (9.5% and 9.5%, respectively). In adult the most reported ORS-like symptoms in the IIV4-I and the IIV4 groups were sore throat (6.7% and 3.4%, respectively) and cough (5.0% and 3.4%, respectively). The non-specific nature of the ORS case definition presents significant limitations. However, the data collected in this study indicates that there is no increase in the incidence of ORS-like symptoms with the use of IIV4-I compared to IIV4.

During the post-vaccination period(s) in infants, the most common injection-site AE was pain, which was reported in about 20% of infants overall, and the most common general AE was fussiness, which was reported in about 30% of infants overall. The most frequent unsolicited AEs were upper respiratory tract illnesses, such as nasopharyngitis and cough. The reactogenicity and safety profile of both vaccines was consistent with previous studies of infants who received GSK’s IIV4s manufactured in Dresden or Québec [9, 16, 18, 38, 39]. In children, the most frequent injection-site AE in both vaccine groups was pain, and the reactogenicity and safety profiles of IIV4-I and IIV4 overall were consistent with previous observations in vaccine-primed and vaccine-unprimed children in the same age range who received IIV4 manufactured in Dresden or Québec, and with children aged 3–8 years in the efficacy study of IIV4 (Québec), versus non-influenza vaccine control [9, 16].

The main limitation of the study was that HI antibody levels are a surrogate outcome for clinical protection against influenza infections, and in children and infants there are no surrogate thresholds defined. However, HI antibody levels were consistent between the IIV4-I and IIV4 vaccines, and both vaccines elicited immune responses at levels considered to be protective in adults [40].

## Conclusion

In conclusion, the study showed that in adults, children, and infants, IIV4-I was immunogenic with a reactogenicity and safety profile that was similar to the licensed IIV4. These results suggest the investigational manufacturing process used to IIV4-I is suitable to replace the current licensed process.

### Trademarks statement

*Influsplit Tetra*, *Fluarix Tetra* and *Fluarix Quadrivalent* are trademarks of the GSK group of companies.

## Additional files

Additional file 1: Solicited oculorespiratory syndrome–like symptoms within the 3-day post vaccination period in infants (6–35 months), children (3–17 years), and adults (18–49 years) (total vaccinated cohort). (PDF 378 kb)

Additional file 2: Serious adverse events in infants (6–35 months), children (3–17 years), and adults (18–49 years) during the entire study (total vaccinated cohort). (PDF 362 kb)

Additional file 3: Medically-attended adverse events in and adults (18–49 years), children (3–17 years), and infants (6–35 months), during the entire study (total vaccinated cohort). (PDF 445 kb)

## Abbreviations

AE: Solicited injection-site adverse event
CBER: Drug Administration Center for Biologics Evaluation and Research
CI: Confidence interval
GI: Gastrointestinal
GMT: Geometric mean titres
HI: Hemagglutination-inhibition
IIV3: Inactivated trivalent influenza vaccine
IIV4: Inactivated quadrivalent influenza vaccine
IIV4-I: Inactivated quadrivalent influenza vaccine investigational
iSRC: Internal Safety Review Committee
MAE: Medically-attended adverse events
ORS: Oculorespiratory syndrome
RR: Relative risk
SAE: Serious adverse events
SCR: Seroconversion rate
SPR: Seroprotection rate
TVC: Total vaccinated cohort
WHO: World Health Organization

## References

[1] United States Centers for Disease Control and Prevention.: Seasonal influenza activity surveillance reports: 2000-2001 to 2010-2011 seasons. http://www.cdc.gov/flu/weekly/pastreports.htm. Accessed August 2017.

[2] Belongia EA, Kieke BA, Donahue JG, Greenlee RT, Balish A, Foust A, Lindstrom S, Shay DK (2009). Effectiveness of inactivated influenza vaccines varied substantially with antigenic match from the 2004-2005 season to the 2006-2007 season. J Infect Dis.

[3] Belshe RB, Coelingh K, Ambrose CS, Woo JC, Wu X (2010). Efficacy of live attenuated influenza vaccine in children against influenza B viruses by lineage and antigenic similarity. Vaccine.

[4] World Health Organization.: Recommended composition of influenza virus vaccines for use in the 2012-2013 northern hemisphere influenza season. 2012, http://www.who.int/influenza/vaccines/virus/recommendations/201202_recommendation.pdf. Accessed August 2017.

[5] Beran J, Peeters M, Dewe W, Raupachova J, Hobzova L, Devaster JM (2013). Immunogenicity and safety of quadrivalent versus trivalent inactivated influenza vaccine: a randomized, controlled trial in adults. BMC Infect Dis.

[6] Block SL, Falloon J, Hirschfield JA, Krilov LR, Dubovsky F, Yi T, Belshe RB (2012). Immunogenicity and safety of a quadrivalent live attenuated influenza vaccine in children. Pediatr Infect Dis J.

[7] Block SL, Yi T, Sheldon E, Dubovsky F, Falloon J (2011). A randomized, double-blind noninferiority study of quadrivalent live attenuated influenza vaccine in adults. Vaccine.

[8] Cadorna-Carlos JB, Nolan T, Borja-Tabora CF, Santos J, Montalban MC, de Looze FJ, Eizenberg P, Hall S, Dupuy M, Hutagalung Y (2015). Safety, immunogenicity, and lot-to-lot consistency of a quadrivalent inactivated influenza vaccine in children, adolescents, and adults: a randomized, controlled, phase III trial. Vaccine.

[9] Domachowske JB, Pankow-Culot H, Bautista M, Feng Y, Claeys C, Peeters M, Innis BL, Jain V (2013). A randomized trial of candidate inactivated quadrivalent influenza vaccine versus trivalent influenza vaccines in children aged 3-17 years. J Infect Dis.

[10] Gorse GJ, Falsey AR, Ozol-Godfrey A, Landolfi V, Tsang PH (2015). Safety and immunogenicity of a quadrivalent intradermal influenza vaccine in adults. Vaccine.

[11] Greenberg DP, Robertson CA, Landolfi VA, Bhaumik A, Senders SD, Decker MD (2014). Safety and immunogenicity of an inactivated quadrivalent influenza vaccine in children 6 months through 8 years of age. Pediatr Infect Dis J.

[12] Greenberg DP, Robertson CA, Noss MJ, Blatter MM, Biedenbender R, Decker MD (2013). Safety and immunogenicity of a quadrivalent inactivated influenza vaccine compared to licensed trivalent inactivated influenza vaccines in adults. Vaccine.

[13] Haber P, Moro PL, Cano M, Lewis P, Stewart B, Shimabukuro TT (2015). Post-licensure surveillance of quadrivalent live attenuated influenza vaccine United States, vaccine adverse event reporting system (VAERS), July 2013-June 2014. Vaccine.

[14] Jain VK, Chandrasekaran V, Wang L, Li P, Liu A, Innis BL (2014). A historically-controlled phase III study in adults to characterize the acceptability of a process change for manufacturing inactivated quadrivalent influenza vaccine. BMC Infect Dis.

[15] Kieninger D, Sheldon E, Lin WY, Yu CJ, Bayas JM, Gabor JJ, Esen M, Fernandez Roure JL, Narejos Perez S, Alvarez Sanchez C (2013). Immunogenicity, reactogenicity and safety of an inactivated quadrivalent influenza vaccine candidate versus inactivated trivalent influenza vaccine: a phase III, randomized trial in adults aged >/=18 years. BMC Infect Dis.

[16] Langley JM, Carmona Martinez A, Chatterjee A, Halperin SA, McNeil S, Reisinger KS, Aggarwal N, Huang LM, Peng CT, Garcia-Sicilia J (2013). Immunogenicity and safety of an inactivated quadrivalent influenza vaccine candidate: a phase III randomized controlled trial in children. J Infect Dis.

[17] Pepin S, Donazzolo Y, Jambrecina A, Salamand C, Saville M (2013). Safety and immunogenicity of a quadrivalent inactivated influenza vaccine in adults. Vaccine.

[18] Rodriguez Weber MA, Claeys C, Aranza Doniz C, Feng Y, Innis BL, Jain VK, Peeters M (2014). Immunogenicity and safety of inactivated quadrivalent and trivalent influenza vaccines in children 18-47 months of age. Pediatr Infect Dis J.

[19] Sheldon EA, Jeanfreau R, Sliman JA, Charenkavanich S, Rousculp MD, Dubovsky F, Mallory RM (2013). Immunogenicity of a quadrivalent Ann Arbor strain live attenuated influenza vaccine delivered using a blow-fill-seal device in adults: a randomized, active-controlled study*. Influenza Other Respir Viruses.

[20] Tinoco JC, Pavia-Ruz N, Cruz-Valdez A, Aranza Doniz C, Chandrasekaran V, Dewe W, Liu A, Innis BL, Jain VK (2014). Immunogenicity, reactogenicity, and safety of inactivated quadrivalent influenza vaccine candidate versus inactivated trivalent influenza vaccine in healthy adults aged >/=18 years: a phase III, randomized trial. Vaccine.

[21] Tsurudome Y, Kimachi K, Okada Y, Matsuura K, Ooyama Y, Ibaragi K, Kino Y, Ueda K. Immunogenicity and safety of an inactivated quadrivalent influenza vaccine in healthy adults: a phase II, open-label, uncontrolled trial in Japan. Microbiol Immunol. 2015. 10.1111/1348-0421.12316 26272602

[22] Jain VK, Rivera L, Zaman K, Espos RA, Sirivichayakul C, Quiambao BP, Rivera-Medina DM, Kerdpanich P, Ceyhan M, Dinleyici EC (2013). Vaccine for prevention of mild and moderate-to-severe influenza in children. N Engl J Med.

[23] Claeys C, Zaman K, Dbaibo G, Li P, Izu A, Kosalaraksa P, Rivera L, Acosta B, Arroba Basanta ML, Aziz A, et al. Prevention of vaccine-matched and mismatched influenza in children 6−35 months of age: a multinational randomized trial across five influenza seasons. Lancet Child Adolescent Health. 2018; in press. 10.1016/S2352-4642(18)30062-2 30169267

[24] United States Centers for Disease Control and Prevention.: Influenza Vaccines — United States, 2014–15 Influenza Season. November 2014, http://www.cdc.gov/flu/protect/vaccine/vaccines.htm. Accessed August 2017.

[25] European Centre for Disease Prevention and Control.: Influenza and vaccination. http://ecdc.europa.eu/en/healthtopics/seasonal_influenza/vaccines/Pages/influenza_vaccination.aspx. Accessed August 2017.

[26] Public Health Agency of Canada National Advisory Committee on Immunization.: Statement on Seasonal Influenza Vaccine for 2014-2015 July 2014, http://www.phac-aspc.gc.ca/naci-ccni/flu-grippe-eng.php. Accessed August 2017.

[27] Australian Government Therapeutic Drugs Administration.: 2015 seasonal influenza vaccines. https://www.tga.gov.au/media-release/2015-seasonal-influenza-vaccines July 2014. Accessed August 2017.

[28] World Health Organization.: Recommended composition of influenza virus vaccines for use in the 2014-2015 northern hemisphere influenza season. 20 February 2014, http://www.who.int/influenza/vaccines/virus/recommendations/2014_15_north/en/. Accessed August 2017.

[29] Grohskopf LA, Olsen SJ, Sokolow LZ, Bresee JS, Cox NJ, Broder KR, Karron RA, Walter EB (2014). Prevention and control of seasonal influenza with vaccines: recommendations of the advisory committee on immunization practices (ACIP) -- United States, 2014-15 influenza season. MMWR Morb Mortal Wkly Rep.

[30] Hehme N, Künzel W, Petschke F, Türk G, Raderecht C, van Hoecke C, Sänger R (2002). Ten years of experience with the trivalent split-influenza vaccine, Fluarix™. Clin Drug Invest.

[31] Miettinen O, Nurminen M (1985). Comparative analysis of two rates. Stat Med.

[32] American Academy of Pediatrics (2014). Recommendations for prevention and control of influenza in children, 2014-2015. Pediatrics.

[33] Al-Dabbagh M, Lapphra K, Scheifele DW, Halperin SA, Langley JM, Cho P, Kollmann TR, Li Y, De Serres G, Fortuno ES (2013). Elevated inflammatory mediators in adults with oculorespiratory syndrome following influenza immunization: a public health agency of Canada/Canadian Institutes of Health Research influenza research network study. Clin Vaccine Immunol.

[34] Armstrong PK, Dowse GK, Effler PV, Carcione D, Blyth CC, Richmond PC, Geelhoed GC, Mascaro F, Scully M, Weeramanthri TS (2011). Epidemiological study of severe febrile reactions in young children in Western Australia caused by a 2010 trivalent inactivated influenza vaccine. BMJ Open.

[35] Petousis-Harris H, Poole T, Booy R, Turner N (2011). Fever following administration of two inactivated influenza vaccines--a survey of parents of New Zealand infants and children 5 years of age and under. Vaccine.

[36] Skowronski DM, Strauss B, De Serres G, MacDonald D, Marion SA, Naus M, Patrick DM, Kendall P (2003). Oculo-respiratory syndrome: a new influenza vaccine-associated adverse event?. Clin Infect Dis.

[37] Maraskovsky E, Rockman S, Dyson A, Koernig S, Becher D, Morelli AB, Barnden M, Camuglia S, Bodle J, Vandenberg K *et al*: Scientific investigations into febrile reactions observed in the paediatric population following vaccination with a 2010 southern hemisphere trivalent influenza vaccine. *Vaccine* 2012, 30(51):7400-7406. 10.1016/j.vaccine.2012.09.083 23063831

[38] Langley JM, Wang IM, Aggarwal N, Bueso A, Chandrasekaran V, Cousin L, Halperin SA, Li P, Liu A, McNeil S, et al. Immunogenicity and Reactogenicity of an inactivated Quadrivalent influenza vaccine administered intramuscularly to children 6 to 35 months of age in 2012–2013: a randomized, double-blind, controlled, multicenter, multicountry, clinical trial. J Pediatr Infect Dis. 2014; 10.1093/jpids/piu1098. Epub. 10.1093/jpids/piu098 PMC4554197 26336604

[39] Wang L, Chandrasekaran V, Domachowske J, Li P, Innis B, Jain V. Immunogenicity and safety of an inactivated Quadrivalent influenza vaccine in US children 6–35 months of age during 2013–2014: results from a phase II randomized trial. J Pediatr Infect Dis. 2015; Epub 16 Iuly 2015. 10.1093/jpids/piv041 PMC5407130 26407273

[40] Hobson D, Curry RL, Beare AS, Ward-Gardner A (1972). The role of serum haemagglutination-inhibiting antibody in protection against challenge infection with influenza A2 and B viruses. J Hyg.

