# Supplementary material for: Assessment of an optimized manufacturing process for inactivated quadrivalent influenza vaccine: a phase III, randomized, double-blind, safety and immunogenicity study in children and adults
Source: BMC Infect Dis. 2018 Apr 18;18:186. doi: 10.1186/s12879-018-3079-8 (PMC5907359; doi:10.1186/s12879-018-3079-8)
Supplement: Supplementary file 1 — Solicited oculorespiratory syndrome–like symptoms within the 3-day post vaccination period in infants (6–35 months), children (3–17 years), and adults (18–49 years) (total vaccinated cohort). (PDF 378 kb) [file 12879_2018_3079_MOESM1_ESM.pdf]

Assessment of an optimized manufacturing process for inactivated quadrivalent influenza vaccine: a Phase III, randomized, double-blind, safety and immunogenicity study in children and adults

Additional file 1

Solicited oculorespiratory syndrome–like symptoms within the 3-day post vaccination period in infants (6–35 months), children (3–17 years), and adults (18–49 years) (total vaccinated cohort)

			**IIV4-I**<br>**Infants: N=462**<br>**Children: N=410**<br>**Adults: N=60**<br><br>**% (95% CI)**	**IIV4**<br>**Infants: N=470**<br>**Children: N=410**<br>**Adults: N=59**<br><br>**% (95% CI)**
**Chest tightness**	Infants	All	2.2 (1.0, 3.9)	1.1 (0.3, 2.5)
		Grade 3	0.0 (0.0, 0.8)	0.4 (0.1, 1.5)
	Children	All	0.5 (0.1, 1.8)	1.5 (0.5, 3.2)
		Grade 3	0.0 (0.0, 0.9)	0.0 (0.0, 0.9)
	Adults	All	0.0 (0.0, 6.0)	0.0 (0.0, 6.1)
		Grade 3	0.0 (0.0, 6.0)	0.0 (0.0, 6.1)
**Cough**	Infants	All	15.8 (12.6, 19.5)	18.1 (14.7, 21.9)
		Grade 3	1.7 (0.8, 3.4)	1.3 (0.5, 2.8)
	Children	All	9.5 (6.9, 12.8)	9.5 (6.9, 12.8)
		Grade 3	0.5 (0.1, 1.8)	0.7 (0.2, 2.1)
	Adults	All	5.0 (1.0, 13.9)	3.4 (0.4, 11.7)
		Grade 3	0.0 (0.0, 6.0)	0.0 (0.0, 6.1)
**Difficulty breathing**	Infants	All	6.7 (4.6, 9.4)	5.3 (3.5, 7.8)
		Grade 3	0.4 (0.1, 1.6)	0.6 (0.1, 1.9)
	Children	All	1.2 (0.4, 2.8)	3.2 (1.7, 5.4)
		Grade 3	0.0 (0.0, 0.9)	0.0 (0.0, 0.9)
	Adults	All	0.0 (0.0, 6.0)	0.0 (0.0, 6.1)
		Grade 3	0.0 (0.0, 6.0)	0.0 (0.0, 6.1)
**Hoarseness**	Infants	All	5.0 (3.2, 7.4)	3.6 (2.1, 5.7)
		Grade 3	0.0 (0.0, 0.8)	0.2 (0.0, 1.2)
	Children	All	2.7 (1.3, 4.7)	3.7 (2.1, 6.0)
		Grade 3	0.0 (0.0, 0.9)	0.0 (0.0, 0.9)

	Adults	All	1.7 (0.0, 8.9)	1.7 (0.0, 9.1)
		Grade 3	0.0 (0.0, 6.0)	0.0 (0.0, 6.1)
**Red eyes**	Infants	All	3.9 (2.3, 6.1)	4.0 (2.5, 6.2)
		Grade 3	0.0 (0.0, 0.8)	0.0 (0.0, 0.8)
	Children	All	3.4 (1.9, 5.7)	3.2 (1.7, 5.4)
		Grade 3	0.0 (0.0, 0.9)	0.2 (0.0, 1.4)
	Adults	All	1.7 (0.0, 8.9)	1.7 (0.0, 9.1)
		Grade 3	0.0 (0.0, 6.0)	0.0 (0.0, 6.1)
**Sore throat**	Infants	All	2.6 (1.3, 4.5)	3.6 (2.1, 5.7)
		Grade 3	0.2 (0.0, 1.2)	0.2 (0.0, 1.2)
	Children	All	3.7 (2.1, 6.0)	5.1 (3.2, 7.7)
		Grade 3	0.2 (0.0, 1.4)	0.2 (0.0, 1.4)
	Adults	All	6.7 (1.8, 16.2)	3.4 (0.4, 11.7)
		Grade 3	0.0 (0.0, 6.0)	0.0 (0.0, 6.1)
**Swallowing difficulty**	Infants	All	1.7 (0.8, 3.4)	2.6 (1.3, 4.4)
		Grade 3	0.4 (0.1, 1.6)	0.2 (0.0, 1.2)
	Children	All	1.5 (0.5, 3.2)	1.7 (0.7, 3.5)
		Grade 3	0.0 (0.0, 0.9)	0.2 (0.0, 1.4)
	Adults	All	5.0 (1.0, 13.9)	1.7 (0.0, 9.1)
		Grade 3	0.0 (0.0, 6.0)	0.0 (0.0, 6.1)
**Swelling of the face**	Infants	All	1.9 (0.9, 3.7)	1.5 (0.6, 3.0)
		Grade 3	0.0 (0.0, 0.8)	0.0 (0.0, 0.8)
	Children	All	0.7 (0.2, 2.1)	0.7 (0.2, 2.1)
		Grade 3	0.0 (0.0, 0.9)	0.0 (0.0, 0.9)
	Adults	All	0.0 (0.0, 6.0)	0.0 (0.0, 6.1)
		Grade 3	0.0 (0.0, 6.0)	0.0 (0.0, 6.1)
**Wheezing**	Infants	All	4.1 (2.5, 6.3)	5.5 (3.6, 8.0)
		Grade 3	0.9 (0.2, 2.2)	0.4 (0.1, 1.5)
	Children	All	0.2 (0.0, 1.4)	1.7 (0.7, 3.5)
		Grade 3	0.0 (0.0, 0.9)	0.0 (0.0, 0.9)
	Adults	All	0.0 (0.0, 6.0)	0.0 (0.0, 6.1)
		Grade 3	0.0 (0.0, 6.0)	0.0 (0.0, 6.1)

IIV4-I, quadrivalent inactivated influenza vaccine manufacturing by investigational process; IIV4, licensed quadrivalent inactivated influenza vaccine; N, number of subjects with ≥1 vaccine dose and who returned the diary cards
