# Supplementary material for: Assessment of an optimized manufacturing process for inactivated quadrivalent influenza vaccine: a phase III, randomized, double-blind, safety and immunogenicity study in children and adults
Source: BMC Infect Dis. 2018 Apr 18;18:186. doi: 10.1186/s12879-018-3079-8 (PMC5907359; doi:10.1186/s12879-018-3079-8)
Supplement: Supplementary file 2 — Serious adverse events in infants (6–35 months), children (3–17 years), and adults (18–49 years) during the entire study (total vaccinated cohort). (PDF 362 kb) [file 12879_2018_3079_MOESM2_ESM.pdf]

Assessment of an optimized manufacturing process for inactivated quadrivalent influenza vaccine: a Phase III, randomized, double-blind, safety and immunogenicity study in children and adults

Additional file 2

Serious adverse events in infants (6–35 months), children (3–17 years), and adults (18–49 years) during the entire study† (total vaccinated cohort)

**System organ class**<br>**Preferred Term**	**Adults**<br>**IIV4-I**<br>**N=60**<br>**n; % (95% CI)**	**Adults**<br>**IIV4**<br>**N=60**<br>**n; % (95% CI)**
At least one symptom	1; 1.7 (0.0, 8.9)	1; 1.7 (0.0, 8.9)
Injury, poisoning and procedural complications (10022117)		
Post procedural inflammation (10063101)	0; 0.0 (0.0, 6.0)	1; 1.7 (0.0, 8.9)
Musculoskeletal and connective tissue disorders (10028395)		
Back pain (10003988)	1; 1.7 (0.0, 8.9)	0; 0.0 (0.0, 6.0)
	**Children**<br>**IIV4-I**<br>**N=410**<br>**n; % (95% CI)**	**Children**<br>**IIV4**<br>**N=411**<br>**n; % (95% CI)**
At least one symptom	1; 0.2 (0.0, 1.4)	0; 0.0 (0.0, 0.9)
Infections and infestations (10021881)		
Meningitis viral (10027260)	1; 0.2 (0.0, 1.4)	0; 0.0 (0.0, 0.9)
	**Infants**<br>**IIV4-I**<br>**N=466**<br>**n; % (95% CI)**	**Infants**<br>**IIV4**<br>**N=474**<br>**n; % (95% CI)**
At least one symptom	7; 1.5 (0.6, 3.1)	11; 2.3 (1.2, 4.1)
Infections and infestations (10021881)		
Adenovirus infection (10060931)	0; 0.0 (0.0, 0.8)	1; 0.2 (0.0, 1.2)
Bronchiolitis (10006448)	0; 0.0 (0.0, 0.8)	2; 0.4 (0.1, 1.5)
Bronchitis (10006451)	2; 0.4 (0.1, 1.5)	2; 0.4 (0.1, 1.5)
Bronchopneumonia (10006469)	1; 0.2 (0.0, 1.2)	0; 0.0 (0.0, 0.8)

Epstein-barr virus infection (10015108)	0; 0.0 (0.0, 0.8)	1; 0.2 (0.0, 1.2)
Gastroenteritis (10017888)	3; 0.6 (0.1, 1.9)	2; 0.4 (0.1, 1.5)
Haemophilus infection (10061190)	0; 0.0 (0.0, 0.8)	1; 0.2 (0.0, 1.2)
Otitis media (10033078)	0; 0.0 (0.0, 0.8)	1; 0.2 (0.0, 1.2)
Pneumonia respiratory syncytial viral (10035732)	1; 0.2 (0.0, 1.2)	0; 0.0 (0.0, 0.8)
Pseudocroup (10050187)	0; 0.0 (0.0, 0.8)	1; 0.2 (0.0, 1.2)
Respiratory syncytial virus bronchiolitis (10038718)	0; 0.0 (0.0, 0.8)	1; 0.2 (0.0, 1.2)
Upper respiratory tract infection (10046306)	1; 0.2 (0.0, 1.2)	0; 0.0 (0.0, 0.8)
Viral infection (10047461)	0; 0.0 (0.0, 0.8)	1; 0.2 (0.0, 1.2)
Metabolism and nutrition disorders (10027433)		
Dehydration (10012174)	1; 0.2 (0.0, 1.2)	0; 0.0 (0.0, 0.8)

IIV4-I, quadrivalent inactivated influenza vaccine manufacturing by investigational process; IIV4, licensed quadrivalent inactivated influenza vaccine; SAE, serious adverse event, CI, confidence interval; †including the allowed visit interval of up to 23 days post-vaccination for the adults and up to 42 days post-last vaccination for the children; N, number of subjects with ≥1 vaccine dose, n, number of subjects reporting the event
