# Supplementary material for: Assessment of an optimized manufacturing process for inactivated quadrivalent influenza vaccine: a phase III, randomized, double-blind, safety and immunogenicity study in children and adults
Source: BMC Infect Dis. 2018 Apr 18;18:186. doi: 10.1186/s12879-018-3079-8 (PMC5907359; doi:10.1186/s12879-018-3079-8)
Supplement: Supplementary file 3 — Medically-attended adverse events in and adults (18–49 years), children (3–17 years), and infants (6–35 months), during the entire study (total vaccinated cohort). (PDF 445 kb) [file 12879_2018_3079_MOESM3_ESM.pdf]

Assessment of an optimized manufacturing process for inactivated quadrivalent influenza vaccine: a Phase III, randomized, double-blind, safety and immunogenicity study in children and adults

Additional file 3

Medically-attended adverse events in and adults (18–49 years), children (3–17 years), and infants (6–35 months), during the entire study† (total vaccinated cohort)

**System Organ Class**<br>**Preferred Term**	**Adults**<br>**IIV4-I**<br>**N=466**<br>**n; % (95% CI)**	**Adults**<br>**IIV4**<br>**N=474**<br>**n; % (95% CI)**
At least one symptom	9; 15.0 (7.1, 26.6)	8; 13.3 (5.9, 24.6)
Immune system disorders (10021428)		
Allergy to arthropod bite (10058285)	0; 0.0 (0.0, 6.0)	1; 1.7 (0.0, 8.9)
Hypersensitivity (10020751)	1; 1.7 (0.0, 8.9)	0; 0.0 (0.0, 6.0)
Infections and infestations (10021881)		
Bronchitis (10006451)	1; 1.7 (0.0, 8.9)	1; 1.7 (0.0, 8.9)
Gastroenteritis (10017888)	1; 1.7 (0.0, 8.9)	2; 3.3 (0.4, 11.5)
Nasopharyngitis (10028810)	0; 0.0 (0.0, 6.0)	2; 3.3 (0.4, 11.5)
Otitis media (10033078)	1; 1.7 (0.0, 8.9)	0; 0.0 (0.0, 6.0)
Sinusitis (10040753)	0; 0.0 (0.0, 6.0)	1; 1.7 (0.0, 8.9)
Tonsillitis (10044008)	1; 1.7 (0.0, 8.9)	0; 0.0 (0.0, 6.0)
Injury, poisoning and procedural complications (10022117)		
Arthropod bite (10003399)	0; 0.0 (0.0, 6.0)	1; 1.7 (0.0, 8.9)
Joint injury (10060820)	1; 1.7 (0.0, 8.9)	0; 0.0 (0.0, 6.0)
Post procedural inflammation (10063101)	0; 0.0 (0.0, 6.0)	1; 1.7 (0.0, 8.9)
Musculoskeletal and connective tissue disorders (10028395)		
Back pain (10003988)	1; 1.7 (0.0, 8.9)	1; 1.7 (0.0, 8.9)
Intervertebral disc protrusion (10050296)	1; 1.7 (0.0, 8.9)	0; 0.0 (0.0, 6.0)
Spinal disorder (10061368)	1; 1.7 (0.0, 8.9)	0; 0.0 (0.0, 6.0)
	**Children**<br>**IIV4-I**<br>**N=466**<br>**n; % (95% CI)**	**Children**<br>**IIV4**<br>**N=474**<br>**n; % (95% CI)**
At least one symptom	235; 50.4 (45.8, 55.1)	252; 53.2 (48.6, 57.7)

Blood and lymphatic system disorders (10005329)		
Lymphadenitis (10025188)	1; 0.2 (0.0, 1.2)	0; 0.0 (0.0, 0.8)
Congenital, familial and genetic disorders (10010331)		
Cryptorchism (10011498)	0; 0.0 (0.0, 0.8)	1; 0.2 (0.0, 1.2)
Ear and labyrinth disorders (10013993)		
Ear pain (10014020)	1; 0.2 (0.0, 1.2)	1; 0.2 (0.0, 1.2)
Middle ear inflammation (10065838)	1; 0.2 (0.0, 1.2)	0; 0.0 (0.0, 0.8)
Eye disorders (10015919)		
Conjunctivitis allergic (10010744)	1; 0.2 (0.0, 1.2)	0; 0.0 (0.0, 0.8)
Gastrointestinal disorders (10017947)		
Anal fissure (10002153)	0; 0.0 (0.0, 0.8)	1; 0.2 (0.0, 1.2)
Aphthous stomatitis (10002958)	0; 0.0 (0.0, 0.8)	2; 0.4 (0.1, 1.5)
Constipation (10010774)	1; 0.2 (0.0, 1.2)	1; 0.2 (0.0, 1.2)
Diarrhoea (10012735)	3; 0.6 (0.1, 1.9)	9; 1.9 (0.9, 3.6)
Enteritis (10014866)	2; 0.4 (0.1, 1.5)	2; 0.4 (0.1, 1.5)
Inguinal hernia (10022016)	0; 0.0 (0.0, 0.8)	1; 0.2 (0.0, 1.2)
Stomatitis (10042128)	2; 0.4 (0.1, 1.5)	1; 0.2 (0.0, 1.2)
Teething (10043183)	1; 0.2 (0.0, 1.2)	1; 0.2 (0.0, 1.2)
Tooth disorder (10044034)	1; 0.2 (0.0, 1.2)	2; 0.4 (0.1, 1.5)
Toothache (10044055)	1; 0.2 (0.0, 1.2)	3; 0.6 (0.1, 1.8)
Umbilical hernia (10045458)	1; 0.2 (0.0, 1.2)	0; 0.0 (0.0, 0.8)
Vomiting (10047700)	4; 0.9 (0.2, 2.2)	6; 1.3 (0.5, 2.7)
General disorders and administration site conditions (10018065)		
Inflammation (10061218)	1; 0.2 (0.0, 1.2)	0; 0.0 (0.0, 0.8)
Pain (10033371)	1; 0.2 (0.0, 1.2)	0; 0.0 (0.0, 0.8)
Pyrexia (10037660)	21; 4.5 (2.8, 6.8)	20; 4.2 (2.6, 6.4)
Immune system disorders (10021428)		
Food allergy (10016946)	0; 0.0 (0.0, 0.8)	1; 0.2 (0.0, 1.2)
Hypersensitivity (10020751)	0; 0.0 (0.0, 0.8)	1; 0.2 (0.0, 1.2)
Infections and infestations (10021881)		
Acarodermatitis (10063409)	1; 0.2 (0.0, 1.2)	0; 0.0 (0.0, 0.8)
Acute tonsillitis (10001093)	0; 0.0 (0.0, 0.8)	2; 0.4 (0.1, 1.5)
Adenovirus infection (10060931)	0; 0.0 (0.0, 0.8)	1; 0.2 (0.0, 1.2)
Bronchiolitis (10006448)	7; 1.5 (0.6, 3.1)	9; 1.9 (0.9, 3.6)
Bronchitis (10006451)	46; 9.9 (7.3, 12.9)	57; 12.0 (9.2, 15.3)
Bronchitis viral (10053160)	0; 0.0 (0.0, 0.8)	1; 0.2 (0.0, 1.2)
Bronchopneumonia (10006469)	6; 1.3 (0.5, 2.8)	1; 0.2 (0.0, 1.2)
Candida infection (10074170)	2; 0.4 (0.1, 1.5)	0; 0.0 (0.0, 0.8)
Candida nappy rash (10007135)	0; 0.0 (0.0, 0.8)	1; 0.2 (0.0, 1.2)
Conjunctivitis (10010741)	17; 3.6 (2.1, 5.8)	16; 3.4 (1.9, 5.4)
Croup infectious (10011416)	2; 0.4 (0.1, 1.5)	0; 0.0 (0.0, 0.8)
Dysentery (10051402)	0; 0.0 (0.0, 0.8)	2; 0.4 (0.1, 1.5)
Ear infection (10014011)	11; 2.4 (1.2, 4.2)	15; 3.2 (1.8, 5.2)
Enterobiasis (10014881)	0; 0.0 (0.0, 0.8)	1; 0.2 (0.0, 1.2)
Epstein-barr virus infection (10015108)	0; 0.0 (0.0, 0.8)	1; 0.2 (0.0, 1.2)
Exanthema subitum (10015586)	1; 0.2 (0.0, 1.2)	0; 0.0 (0.0, 0.8)
Febrile infection (10051998)	1; 0.2 (0.0, 1.2)	0; 0.0 (0.0, 0.8)
Fungal infection (10017533)	2; 0.4 (0.1, 1.5)	0; 0.0 (0.0, 0.8)
Fungal skin infection (10017543)	2; 0.4 (0.1, 1.5)	1; 0.2 (0.0, 1.2)

Gastroenteritis (10017888)	29; 6.2 (4.2, 8.8)	34; 7.2 (5.0, 9.9)
Gastroenteritis viral (10017918)	1; 0.2 (0.0, 1.2)	0; 0.0 (0.0, 0.8)
Gianotti-crosti syndrome (10053842)	0; 0.0 (0.0, 0.8)	1; 0.2 (0.0, 1.2)
Giardiasis (10018262)	1; 0.2 (0.0, 1.2)	0; 0.0 (0.0, 0.8)
Gingivitis (10018292)	0; 0.0 (0.0, 0.8)	1; 0.2 (0.0, 1.2)
Haemophilus infection (10061190)	0; 0.0 (0.0, 0.8)	1; 0.2 (0.0, 1.2)
Hand-foot-and-mouth disease (10019113)	3; 0.6 (0.1, 1.9)	5; 1.1 (0.3, 2.4)
Herpangina (10019936)	4; 0.9 (0.2, 2.2)	4; 0.8 (0.2, 2.1)
Herpes virus infection (10019973)	1; 0.2 (0.0, 1.2)	1; 0.2 (0.0, 1.2)
Hordeolum (10020377)	1; 0.2 (0.0, 1.2)	1; 0.2 (0.0, 1.2)
Impetigo (10021531)	1; 0.2 (0.0, 1.2)	1; 0.2 (0.0, 1.2)
Laryngitis (10023874)	3; 0.6 (0.1, 1.9)	8; 1.7 (0.7, 3.3)
Lice infestation (10024424)	1; 0.2 (0.0, 1.2)	0; 0.0 (0.0, 0.8)
Lower respiratory tract infection (10024968)	5; 1.1 (0.3, 2.5)	1; 0.2 (0.0, 1.2)
Molluscum contagiosum (10027807)	0; 0.0 (0.0, 0.8)	1; 0.2 (0.0, 1.2)
Myringitis (10061302)	0; 0.0 (0.0, 0.8)	1; 0.2 (0.0, 1.2)
Nasopharyngitis (10028810)	27; 5.8 (3.9, 8.3)	30; 6.3 (4.3, 8.9)
Oral candidiasis (10030963)	1; 0.2 (0.0, 1.2)	2; 0.4 (0.1, 1.5)
Oral fungal infection (10061324)	0; 0.0 (0.0, 0.8)	1; 0.2 (0.0, 1.2)
Oral herpes (10067152)	2; 0.4 (0.1, 1.5)	1; 0.2 (0.0, 1.2)
Otitis externa (10033072)	0; 0.0 (0.0, 0.8)	1; 0.2 (0.0, 1.2)
Otitis media (10033078)	8; 1.7 (0.7, 3.4)	8; 1.7 (0.7, 3.3)
Otitis media acute (10033079)	19; 4.1 (2.5, 6.3)	14; 3.0 (1.6, 4.9)
Pharyngitis (10034835)	13; 2.8 (1.5, 4.7)	7; 1.5 (0.6, 3.0)
Pharyngotonsillitis (10049140)	3; 0.6 (0.1, 1.9)	3; 0.6 (0.1, 1.8)
Pneumonia (10035664)	3; 0.6 (0.1, 1.9)	4; 0.8 (0.2, 2.1)
Pneumonia respiratory syncytial viral (10035732)	1; 0.2 (0.0, 1.2)	0; 0.0 (0.0, 0.8)
Pseudocroup (10050187)	1; 0.2 (0.0, 1.2)	1; 0.2 (0.0, 1.2)
Rash pustular (10037888)	1; 0.2 (0.0, 1.2)	0; 0.0 (0.0, 0.8)
Respiratory syncytial virus bronchiolitis (10038718)	0; 0.0 (0.0, 0.8)	1; 0.2 (0.0, 1.2)
Respiratory tract infection (10062352)	6; 1.3 (0.5, 2.8)	7; 1.5 (0.6, 3.0)
Respiratory tract infection viral (10062106)	2; 0.4 (0.1, 1.5)	5; 1.1 (0.3, 2.4)
Rhinitis (10039083)	6; 1.3 (0.5, 2.8)	9; 1.9 (0.9, 3.6)
Rhinotracheitis (10051497)	3; 0.6 (0.1, 1.9)	5; 1.1 (0.3, 2.4)
Roseola (10039222)	2; 0.4 (0.1, 1.5)	5; 1.1 (0.3, 2.4)
Scarlet fever (10039587)	0; 0.0 (0.0, 0.8)	1; 0.2 (0.0, 1.2)
Sinobronchitis (10048979)	0; 0.0 (0.0, 0.8)	1; 0.2 (0.0, 1.2)
Sinusitis (10040753)	2; 0.4 (0.1, 1.5)	0; 0.0 (0.0, 0.8)
Streptococcal infection (10061372)	0; 0.0 (0.0, 0.8)	1; 0.2 (0.0, 1.2)
Tonsillitis (10044008)	6; 1.3 (0.5, 2.8)	7; 1.5 (0.6, 3.0)
Tracheitis (10044302)	6; 1.3 (0.5, 2.8)	8; 1.7 (0.7, 3.3)
Upper respiratory tract infection (10046306)	60; 12.9 (10.0, 16.3)	66; 13.9 (10.9, 17.4)
Urinary tract infection (10046571)	2; 0.4 (0.1, 1.5)	2; 0.4 (0.1, 1.5)
Varicella (10046980)	2; 0.4 (0.1, 1.5)	4; 0.8 (0.2, 2.1)
Viral infection (10047461)	10; 2.1 (1.0, 3.9)	15; 3.2 (1.8, 5.2)
Viral rash (10047476)	2; 0.4 (0.1, 1.5)	2; 0.4 (0.1, 1.5)
Viral tonsillitis (10047480)	0; 0.0 (0.0, 0.8)	1; 0.2 (0.0, 1.2)
Injury, poisoning and procedural complications (10022117)		
Arthropod bite (10003399)	3; 0.6 (0.1, 1.9)	1; 0.2 (0.0, 1.2)
Contusion (10050584)	0; 0.0 (0.0, 0.8)	1; 0.2 (0.0, 1.2)
Face injury (10050392)	1; 0.2 (0.0, 1.2)	0; 0.0 (0.0, 0.8)
Foreign body (10070245)	1; 0.2 (0.0, 1.2)	0; 0.0 (0.0, 0.8)
Hair injury (10074924)	0; 0.0 (0.0, 0.8)	1; 0.2 (0.0, 1.2)
Head injury (10019196)	1; 0.2 (0.0, 1.2)	1; 0.2 (0.0, 1.2)
Injury (10022116)	0; 0.0 (0.0, 0.8)	1; 0.2 (0.0, 1.2)

Joint dislocation (10023204)	0; 0.0 (0.0, 0.8)	1; 0.2 (0.0, 1.2)
Limb injury (10061225)	1; 0.2 (0.0, 1.2)	0; 0.0 (0.0, 0.8)
Lip injury (10055082)	1; 0.2 (0.0, 1.2)	0; 0.0 (0.0, 0.8)
Thermal burn (10053615)	2; 0.4 (0.1, 1.5)	0; 0.0 (0.0, 0.8)
Traumatic haematoma (10044522)	1; 0.2 (0.0, 1.2)	0; 0.0 (0.0, 0.8)
Wound dehiscence (10048031)	0; 0.0 (0.0, 0.8)	1; 0.2 (0.0, 1.2)
Metabolism and nutrition disorders (10027433)		
Dehydration (10012174)	1; 0.2 (0.0, 1.2)	0; 0.0 (0.0, 0.8)
Iron deficiency (10022970)	1; 0.2 (0.0, 1.2)	0; 0.0 (0.0, 0.8)
Lactose intolerance (10023681)	1; 0.2 (0.0, 1.2)	0; 0.0 (0.0, 0.8)
Weight gain poor (10047897)	0; 0.0 (0.0, 0.8)	1; 0.2 (0.0, 1.2)
Musculoskeletal and connective tissue disorders (10028395)		
Synovitis (10042868)	1; 0.2 (0.0, 1.2)	1; 0.2 (0.0, 1.2)
Neoplasms benign, malignant and unspecified (10029104)		
Melanocytic naevus (10027145)	0; 0.0 (0.0, 0.8)	1; 0.2 (0.0, 1.2)
Nervous system disorders (10029205)		
Febrile convulsion (10016284)	0; 0.0 (0.0, 0.8)	1; 0.2 (0.0, 1.2)
Psychiatric disorders (10037175)		
Bruxism (10006514)	1; 0.2 (0.0, 1.2)	0; 0.0 (0.0, 0.8)
Insomnia (10022437)	0; 0.0 (0.0, 0.8)	1; 0.2 (0.0, 1.2)
Irritability (10022998)	1; 0.2 (0.0, 1.2)	1; 0.2 (0.0, 1.2)
Sleep disorder (10040984)	1; 0.2 (0.0, 1.2)	0; 0.0 (0.0, 0.8)
Reproductive system and breast disorders (10038604)		
Balanoposthitis (10004078)	2; 0.4 (0.1, 1.5)	0; 0.0 (0.0, 0.8)
Respiratory, thoracic and mediastinal disorders (10038738)		
Allergic bronchitis (10052613)	1; 0.2 (0.0, 1.2)	0; 0.0 (0.0, 0.8)
Allergic cough (10053779)	0; 0.0 (0.0, 0.8)	2; 0.4 (0.1, 1.5)
Asthma (10003553)	4; 0.9 (0.2, 2.2)	3; 0.6 (0.1, 1.8)
Asthmatic crisis (10064823)	1; 0.2 (0.0, 1.2)	0; 0.0 (0.0, 0.8)
Atelectasis (10003598)	0; 0.0 (0.0, 0.8)	1; 0.2 (0.0, 1.2)
Bronchial hyperreactivity (10066091)	5; 1.1 (0.3, 2.5)	7; 1.5 (0.6, 3.0)
Bronchospasm (10006482)	11; 2.4 (1.2, 4.2)	9; 1.9 (0.9, 3.6)
Cough (10011224)	11; 2.4 (1.2, 4.2)	14; 3.0 (1.6, 4.9)
Epistaxis (10015090)	0; 0.0 (0.0, 0.8)	1; 0.2 (0.0, 1.2)
Oropharyngeal pain (10068319)	1; 0.2 (0.0, 1.2)	1; 0.2 (0.0, 1.2)
Rhinitis allergic (10039085)	0; 0.0 (0.0, 0.8)	1; 0.2 (0.0, 1.2)
Rhinorrhoea (10039101)	3; 0.6 (0.1, 1.9)	1; 0.2 (0.0, 1.2)
Skin and subcutaneous tissue disorders (10040785)		
Dermatitis (10012431)	4; 0.9 (0.2, 2.2)	1; 0.2 (0.0, 1.2)
Dermatitis allergic (10012434)	2; 0.4 (0.1, 1.5)	1; 0.2 (0.0, 1.2)
Dermatitis atopic (10012438)	0; 0.0 (0.0, 0.8)	2; 0.4 (0.1, 1.5)
Dermatitis diaper (10012444)	7; 1.5 (0.6, 3.1)	3; 0.6 (0.1, 1.8)
Dry skin (10013786)	1; 0.2 (0.0, 1.2)	0; 0.0 (0.0, 0.8)
Eczema (10014184)	6; 1.3 (0.5, 2.8)	4; 0.8 (0.2, 2.1)
Idiopathic urticaria (10021247)	0; 0.0 (0.0, 0.8)	1; 0.2 (0.0, 1.2)
Nail dystrophy (10028698)	0; 0.0 (0.0, 0.8)	1; 0.2 (0.0, 1.2)
Neurodermatitis (10029263)	1; 0.2 (0.0, 1.2)	0; 0.0 (0.0, 0.8)
Rash (10037844)	2; 0.4 (0.1, 1.5)	7; 1.5 (0.6, 3.0)

Skin fissures (10040849)	1; 0.2 (0.0, 1.2)	0; 0.0 (0.0, 0.8)
Skin hyperpigmentation (10040865)	0; 0.0 (0.0, 0.8)	1; 0.2 (0.0, 1.2)
Urticaria (10046735)	2; 0.4 (0.1, 1.5)	1; 0.2 (0.0, 1.2)
	**Infants IIV4-I N=410 n; % (95% CI)**	**Infants IIV4 N=411 n; % (95% CI)**
At least one symptom	59; 14.4 (11.1, 18.2)	52; 12.7 (9.6, 16.3)
Blood and lymphatic system disorders (10005329)		
Lymphadenitis (10025188)	1; 0.2 (0.0, 1.4)	0; 0.0 (0.0, 0.9)
Lymphadenopathy (10025197)	1; 0.2 (0.0, 1.4)	0; 0.0 (0.0, 0.9)
Ear and labyrinth disorders (10013993)		
Otorrhoea (10033101)	0; 0.0 (0.0, 0.9)	1; 0.2 (0.0, 1.3)
Gastrointestinal disorders (10017947)		
Abdominal pain (10000081)	2; 0.5 (0.1, 1.8)	0; 0.0 (0.0, 0.9)
Constipation (10010774)	1; 0.2 (0.0, 1.4)	1; 0.2 (0.0, 1.3)
Inguinal hernia (10022016)	0; 0.0 (0.0, 0.9)	1; 0.2 (0.0, 1.3)
Oral pain (10031009)	0; 0.0 (0.0, 0.9)	1; 0.2 (0.0, 1.3)
Stomatitis (10042128)	0; 0.0 (0.0, 0.9)	1; 0.2 (0.0, 1.3)
Vomiting (10047700)	2; 0.5 (0.1, 1.8)	0; 0.0 (0.0, 0.9)
General disorders and administration site conditions (10018065)		
Chest pain (10008479)	0; 0.0 (0.0, 0.9)	1; 0.2 (0.0, 1.3)
Pyrexia (10037660)	1; 0.2 (0.0, 1.4)	1; 0.2 (0.0, 1.3)
Immune system disorders (10021428)		
Food allergy (10016946)	0; 0.0 (0.0, 0.9)	1; 0.2 (0.0, 1.3)
Milk allergy (10027633)	0; 0.0 (0.0, 0.9)	1; 0.2 (0.0, 1.3)
Infections and infestations (10021881)		
Acute tonsillitis (10001093)	1; 0.2 (0.0, 1.4)	1; 0.2 (0.0, 1.3)
Bronchitis (10006451)	7; 1.7 (0.7, 3.5)	4; 1.0 (0.3, 2.5)
Bronchopneumonia (10006469)	1; 0.2 (0.0, 1.4)	0; 0.0 (0.0, 0.9)
Cellulitis (10007882)	7; 1.7 (0.7, 3.5)	4; 1.0 (0.3, 2.5)
Conjunctivitis (10010741)	1; 0.2 (0.0, 1.4)	0; 0.0 (0.0, 0.9)
Cystitis (10011781)	0; 0.0 (0.0, 0.9)	1; 0.2 (0.0, 1.3)
Ear infection (10014011)	1; 0.2 (0.0, 1.4)	0; 0.0 (0.0, 0.9)
Enterovirus infection (10014909)	0; 0.0 (0.0, 0.9)	1; 0.2 (0.0, 1.3)
Gastroenteritis (10017888)	0; 0.0 (0.0, 0.9)	3; 0.7 (0.2, 2.1)
Herpangina (10019936)	1; 0.2 (0.0, 1.4)	0; 0.0 (0.0, 0.9)
Herpes simplex (10019948)	1; 0.2 (0.0, 1.4)	0; 0.0 (0.0, 0.9)
Impetigo (10021531)	1; 0.2 (0.0, 1.4)	5; 1.2 (0.4, 2.8)
Laryngitis (10023874)	0; 0.0 (0.0, 0.9)	1; 0.2 (0.0, 1.3)
Meningitis viral (10027260)	1; 0.2 (0.0, 1.4)	1; 0.2 (0.0, 1.3)
Molluscum contagiosum (10027807)	2; 0.5 (0.1, 1.8)	0; 0.0 (0.0, 0.9)
Nasopharyngitis (10028810)	2; 0.5 (0.1, 1.8)	2; 0.5 (0.1, 1.7)
Oral herpes (10067152)	0; 0.0 (0.0, 0.9)	2; 0.5 (0.1, 1.7)
Otitis externa (10033072)	1; 0.2 (0.0, 1.4)	0; 0.0 (0.0, 0.9)
Otitis media (10033078)	2; 0.5 (0.1, 1.8)	1; 0.2 (0.0, 1.3)

Otitis media acute (10033079)	2; 0.5 (0.1, 1.8)	1; 0.2 (0.0, 1.3)
Paronychia (10034016)	1; 0.2 (0.0, 1.4)	0; 0.0 (0.0, 0.9)
Periporitis staphylogenes (10064163)	1; 0.2 (0.0, 1.4)	0; 0.0 (0.0, 0.9)
Pertussis (10034738)	1; 0.2 (0.0, 1.4)	0; 0.0 (0.0, 0.9)
Pharyngitis (10034835)	6; 1.5 (0.5, 3.2)	4; 1.0 (0.3, 2.5)
Pharyngitis streptococcal (10034839)	2; 0.5 (0.1, 1.8)	0; 0.0 (0.0, 0.9)
Pharyngotonsillitis (10049140)	2; 0.5 (0.1, 1.8)	2; 0.5 (0.1, 1.7)
Pseudocroup (10050187)	1; 0.2 (0.0, 1.4)	0; 0.0 (0.0, 0.9)
Respiratory tract infection (10062352)	0; 0.0 (0.0, 0.9)	1; 0.2 (0.0, 1.3)
Rhinitis (10039083)	1; 0.2 (0.0, 1.4)	5; 1.2 (0.4, 2.8)
Scarlet fever (10039587)	0; 0.0 (0.0, 0.9)	1; 0.2 (0.0, 1.3)
Sinusitis (10040753)	1; 0.2 (0.0, 1.4)	2; 0.5 (0.1, 1.7)
Tonsillitis (10044008)	3; 0.7 (0.2, 2.1)	2; 0.5 (0.1, 1.7)
Tooth abscess (10044016)	1; 0.2 (0.0, 1.4)	0; 0.0 (0.0, 0.9)
Tracheitis (10044302)	2; 0.5 (0.1, 1.8)	2; 0.5 (0.1, 1.7)
Upper respiratory tract infection (10046306)	4; 1.0 (0.3, 2.5)	8; 1.9 (0.8, 3.8)
Viral diarrhoea (10051511)	0; 0.0 (0.0, 0.9)	1; 0.2 (0.0, 1.3)
Viral infection (10047461)	3; 0.7 (0.2, 2.1)	0; 0.0 (0.0, 0.9)
Vulvitis (10047780)	1; 0.2 (0.0, 1.4)	0; 0.0 (0.0, 0.9)
Injury, poisoning and procedural complications (10022117)		
Arthropod bite (10003399)	1; 0.2 (0.0, 1.4)	1; 0.2 (0.0, 1.3)
Contusion (10050584)	1; 0.2 (0.0, 1.4)	1; 0.2 (0.0, 1.3)
Head injury (10019196)	0; 0.0 (0.0, 0.9)	1; 0.2 (0.0, 1.3)
Joint injury (10060820)	1; 0.2 (0.0, 1.4)	0; 0.0 (0.0, 0.9)
Laceration (10023572)	1; 0.2 (0.0, 1.4)	1; 0.2 (0.0, 1.3)
Ligament sprain (10024453)	0; 0.0 (0.0, 0.9)	1; 0.2 (0.0, 1.3)
Wound (10052428)	1; 0.2 (0.0, 1.4)	0; 0.0 (0.0, 0.9)
Musculoskeletal and connective tissue disorders (10028395)		
Neck pain (10028836)	1; 0.2 (0.0, 1.4)	0; 0.0 (0.0, 0.9)
Neoplasms benign, malignant and unspecified (10029104)		
Skin papilloma (10040907)	1; 0.2 (0.0, 1.4)	0; 0.0 (0.0, 0.9)
Nervous system disorders (10029205)		
Headache (10019211)	0; 0.0 (0.0, 0.9)	1; 0.2 (0.0, 1.3)
Syncope (10042772)	1; 0.2 (0.0, 1.4)	1; 0.2 (0.0, 1.3)
Renal and urinary disorders (10038359)		
Enuresis (10014928)	1; 0.2 (0.0, 1.4)	0; 0.0 (0.0, 0.9)
Respiratory, thoracic and mediastinal disorders (10038738)		
Bronchial hyperreactivity (10066091)	1; 0.2 (0.0, 1.4)	1; 0.2 (0.0, 1.3)
Bronchospasm (10006482)	0; 0.0 (0.0, 0.9)	1; 0.2 (0.0, 1.3)
Cough (10011224)	4; 1.0 (0.3, 2.5)	4; 1.0 (0.3, 2.5)
Dyspnoea (10013968)	1; 0.2 (0.0, 1.4)	0; 0.0 (0.0, 0.9)
Oropharyngeal pain (10068319)	0; 0.0 (0.0, 0.9)	2; 0.5 (0.1, 1.7)
Tonsillar hypertrophy (10044003)	0; 0.0 (0.0, 0.9)	1; 0.2 (0.0, 1.3)
Skin and subcutaneous tissue disorders (10040785)		
Dermatitis (10012431)	1; 0.2 (0.0, 1.4)	2; 0.5 (0.1, 1.7)
Dry skin (10013786)	1; 0.2 (0.0, 1.4)	0; 0.0 (0.0, 0.9)
Eczema (10014184)	1; 0.2 (0.0, 1.4)	0; 0.0 (0.0, 0.9)

IIV4-I, quadrivalent inactivated influenza vaccine manufacturing by investigational process; IIV4, licensed quadrivalent inactivated influenza vaccine; MAE, medically-attended adverse event; †including the allowed visit interval of up to 23 days post-vaccination for the adults and up to 42 days post-last vaccination for the children; N, number of subjects with ≥1 vaccine dose; n, number of participants reporting the MAE; CI, confidence interval
